# Unconjugated PLGA nanoparticles attenuate temperature-dependent β-amyloid aggregation and protect neurons against toxicity: implications for Alzheimer’s disease pathology

**DOI:** 10.1186/s12951-022-01269-0

**Published:** 2022-02-04

**Authors:** Pallabi Sil Paul, Jae-Young Cho, Qi Wu, Govindarajan Karthivashan, Emily Grabovac, Holger Wille, Mariana Kulka, Satyabrata Kar

**Affiliations:** 1 Department of Medicine (Neurology), Centre for Prions and Protein Folding Diseases, University of Alberta, Edmonton, AB T6G 2M8 Canada; 2 Nanotechnology Research Centre, National Research Council Canada, Edmonton, AB T6G 2M9 Canada; 3 Department of Biochemistry, Centre for Prions and Protein Folding Diseases, University of Alberta, Edmonton, AB T6G 2M8 Canada; 4 Department of Medical Microbiology and Immunology, University of Alberta, Edmonton, AB T6G 2E1 Canada; 5 Departments of Medicine (Neurology) and Psychiatry, Centre for Prions and Protein Folding Diseases, University of Alberta, Edmonton, AB T6G 2M8 Canada

**Keywords:** Alzheimer’s disease, β-amyloid aggregation, PLGA nanoparticles, Neuroprotection

## Abstract

Conversion of β-amyloid (Aβ) peptides from soluble random-coil to aggregated protein enriched with β-sheet-rich intermediates has been suggested to play a role in the degeneration of neurons and development of Alzheimer’s disease (AD) pathology. Aggregation of Aβ peptide can be prompted by a variety of environmental factors including temperature which can influence disease pathogenesis. Recently, we reported that FDA-approved unconjugated poly (d,l-lactide-co-glycolide) (PLGA) nanoparticles can have beneficial effects in cellular and animal models of AD by targeting different facets of the Aβ axis. In this study, using biochemical, structural and spectroscopic analyses, we evaluated the effects of native PLGA on temperature-dependent Aβ aggregation and its ability to protect cultured neurons from degeneration. Our results show that the rate of spontaneous Aβ_1–42_ aggregation increases with a rise in temperature from 27 to 40 °C and PLGA with 50:50 resomer potently inhibits Aβ aggregation at all temperatures, but the effect is more profound at 27 °C than at 40 °C. It appears that native PLGA, by interacting with the hydrophobic domain of Aβ_1–42_, prevents a conformational shift towards β-sheet structure, thus precluding the formation of Aβ aggregates. Additionally, PLGA triggers disassembly of matured Aβ_1–42_ fibers at a faster rate at 40 °C than at 27 °C. PLGA-treated Aβ samples can significantly enhance viability of cortical cultured neurons compared to neurons treated with Aβ alone by attenuating phosphorylation of tau protein. Injection of native PLGA is found to influence the breakdown/clearance of Aβ peptide in the brain. Collectively, these results suggest that PLGA nanoparticles can inhibit Aβ aggregation and trigger disassembly of Aβ aggregates at temperatures outside the physiological range and can protect neurons against Aβ-mediated toxicity thus validating its unique therapeutic potential in the treatment of AD pathology.

**Graphical Abstract:**

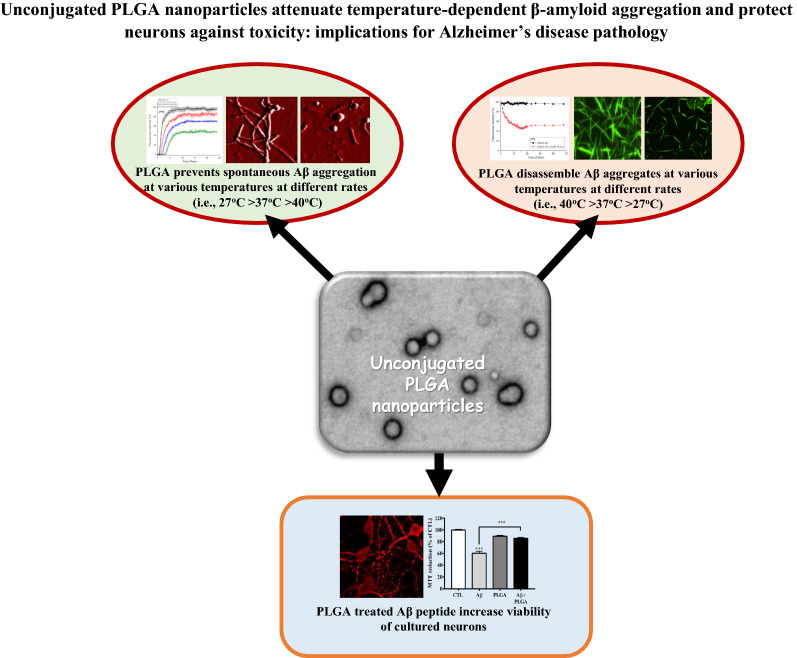

**Supplementary Information:**

The online version contains supplementary material available at 10.1186/s12951-022-01269-0.

## Introduction

Alzheimer’s disease (AD) is an unrelenting neurodegenerative disorder characterized neuropathologically by the presence of tau-positive intracellular neurofibrillary tangles, β-amyloid (Aβ)-containing extracellular neuritic plaques and the loss of synapses/neurons in selected regions of the brain. Etiologically, only a minority (< 10%) of AD cases segregate with mutations of three known genes, whereas the majority of cases are sporadic. Other factors that increase the risk of AD include age, head injury, stress and inheritance of an apolipoprotein E4 genotype [1–3]. Evidence suggests that increased levels of Aβ peptides, generated from amyloid precursor protein, contribute to the loss of neurons and subsequent development of AD pathology. Although various Aβ peptides containing 39–43 amino acids are produced in normal conditions, the two most common isoforms found in the brain are Aβ_1-40_ and Aβ_1–42_, of which Aβ_1–42,_ due to its more hydrophobic nature, tends to aggregate faster and is more toxic to neurons than the Aβ_1–40_ [4–7]. The transition of soluble Aβ monomer to the fibrillar form is often linked with the neurotoxicity mediated via site-specific phosphorylation of tau protein and subsequent progression of AD pathology [1, 3, 8, 9]. While the C-terminal domain of Aβ determines the rate of fibril formation, the N-terminal region fosters Aβ–Aβ polymerization, leading to a random coil or α-helix to β-sheet transition. The extended β-sheet promotes Aβ oligomer formation which serves as nuclei for fibril growth and subsequent deposition of Aβ aggregates in neuritic plaques of AD brains [10, 11]. Over the years, a number of pharmacological and non-pharmacological strategies have been used to interfere with Aβ aggregation/toxicity as potential treatments for AD.

The conformational transition of the Aβ peptide, apart from increased Aβ levels, is known to be influenced by a variety of environmental factors including pH, metal ions, oxidation state, high cholesterol, ionic strength and temperature [12–18]. Temperature, like any other kinetical reaction, facilitates Aβ fibrilization as the velocity of Aβ aggregation at 25 °C is reported to be about 10% of that at 37 °C [19]. Variation in ambient temperatures has also been shown to influence Aβ levels/aggregation and phosphorylation of tau protein in a variety of cellular and animal models of AD [20–24]. Human body temperature, under normal condition, varies in the range from 35 to 39 °C and increases to 42 °C during fever [25–27], whereas local temperature in different brain diseases/tumors fluctuates from 33.4 to 42 °C [18, 28, 29]. A recent prospective study reveals that high-temperature sauna baths can lower the risk of developing dementia and AD pathology in humans [30]. Although these studies indicate that temperature can influence AD-related pathology, very little is known about how temperature can affect Aβ aggregation or kinetic reaction in the presence of small molecular agents known to attenuate Aβ aggregation/toxicity.

Over the last decade, a number of small molecules including nanoparticles, which are engineered materials less than 100 nm in diameter with unique physio-chemical properties, have been explored extensively as novel therapeutic modalities to inhibit Aβ aggregation/toxicity. Intrinsic structural and surface properties of nanoparticles such as size, shape, net-charge and surface hydrophobicity regulate their interactions with Aβ peptides [31–35]. Interestingly, acidic poly (d,l-lactide-co-glycolide) (PLGA) nanoparticles, which constitute a family of FDA-approved biodegradable polymers synthesized from glycolic acid and lactic acid, have long been studied as delivery vehicles for a variety of drugs, proteins and other macromolecules. In fact, some earlier studies have shown that PLGA-encapsulated drugs/agents such as donepezil, memantine etc., can have beneficial effects on cellular and/or animal models of AD with satisfactory biocompatibility [36–41]. More recently, we reported that PLGA nanoparticles without functionalization with any agent/drug can suppress Aβ aggregation/toxicity in cellular and animal models of AD [42, 43]. In the present study, we evaluated how hypothermic (27 °C) and hyperthermic (40 °C) conditions can influence spontaneous Aβ_1–42_ aggregation compared to normal conditions (37 °C). Additionally, we analyzed if unconjugated PLGA can suppress Aβ_1–42_ aggregation/toxicity at hypothermic and hyperthermic conditions as observed in normal condition, thus highlighting the significance of temperature and PLGA in regulating Aβ pathology.

## Materials and methods

### Materials

Recombinant human Aβ_1–42_ peptide (> 97% purity, lyophilized powder) and human Aβ_42–1_ peptide (> 95% purity, lyophilized powder) were purchased from rPeptide R&D (Sunnyvale, CA, USA) and Anaspec (Sunnyvale, CA, USA), respectively. PLGA (50:50 resomer; lactic acid:glycolic acid) was from Phosphorex (Hopkinton, MA, USA). Hexafluoro-2-Propanol (HFIP), Thioflavin T (ThT), dimethyl sulfoxide (DMSO), PLGA (50:50 and 75:25 resomers; lactic acid:glycolic acid), polycaprolactone (PCL), polyethylene glycol (PEG)-PLGA and tetrahydrofuran and 3-(4,5-dimethylthiazol-2-yl)-2,5-diphenyltetrazolium bromide (MTT) were obtained from Sigma-Aldrich (St. Louis Missouri, USA), whereas lactate dehydrogenase (LDH)-based cytotoxicity assay kit was from Promega (Wisconsin, USA). Electron microscopy grids (carbon coated 400 mesh copper grids) and uranyl acid stains were purchased from Electron Microscopy Sciences (PA, USA). Dulbecco’s modified Eagle’s medium (DMEM), neurobasal medium, Hanks’ balanced salt solution (HBSS), fetal bovine serum (FBS) and fluorescein-Aβ_1–42_ were purchased from rPeptide (Watkinsville, USA). Sources of primary antibodies used in the study are listed in Table 1. All horseradish peroxidase-conjugated secondary antibodies were from Bio-Rad Lab (Hercules, CA, USA). All other chemicals were obtained from either Sigma-Aldrich or Thermo Fisher Scientific.

**Table 1:** Details of the primary antibodies used in this study

Antibody type	Type	WB/FT dilution	Source
Amyloid fibrils OC	Polyclonal	1:1000	Sigma-Aldrich
β-actin	Monoclonal	1:5000	Sigma-Aldrich
Oligomer A11	Polyclonal	1:1000	Invitrogen
Phospho-ERK	Polyclonal	1:1000	Cell signaling
Phospho-GSK	Polyclonal	1:1000	Abcam
Total-ERK	Monoclonal	1:1000	Cell signaling
Total-GSK	Monoclonal	1:1000	Abcam
Tau (AT270)	Monoclonal	1:1000	Thermo fisher

*WB* western blotting; *FT* filter-trap assay

### Preparation of Aβ peptides

Lyophilized Aβ_1–42_ and Aβ_42–1_ stored at − 80 °C were first equilibrated at room temperature for 30 min before dissolving in HFIP to obtain a 1 mM solution. Once dissolved, peptide aliquots were quickly dried using a SpeedVac to remove HFIP and then stored at − 80 °C for subsequent analysis as described previously [44]. On the day of experiment, Aβ_1–42_ aliquot was thawed at 4 °C, diluted with DMSO to obtain 5 mM concentration and then with phosphate-buffer saline (0.01 M PBS, pH 7.4) to 100 μM concentration and finally incubated at 4 °C for 22 h to get Aβ oligomers. As for fibril preparation, Aβ_1–42_ aliquot was diluted to 100 μM concentration with 0.01 M PBS (pH 7.4) and then incubated at 37 °C for 24 h to get Aβ fibrils. Both Aβ oligomers and/or fibrils were then used for experiments in presence or absence of unconjugated PLGA, PEG-PLGA and PCL nanoparticles.

### Preparation of PLGA, PEG-PLGA and PCL nanoparticles

PLGA, PEG-PLGA and PCL nanoparticles were prepared following manufacture’s instruction as described previously [42]. In brief, PLGA powder is dissolved into PBS (0.01 M PBS, pH 7.4) followed by sonication using a probe sonicator with 40 pulses and 40% amplitude, whereas PEG-PLGA and PCL were initially dissolved in tetrahydrofuran and then diluted to required concentrations in PBS buffer.

### Aβ aggregation kinetics

The aggregation kinetics at different concentrations (i.e., 2.5–20 μM) of Aβ_1–42_ were carried out in 150 μl reaction buffer containing 10 mM Na_2_HPO_4_ with 100 mM NaCl (pH 7.4) at 27 °C, 37 °C and 40 °C in the absence or presence of different concentrations (5 μM, 10 μM and 25 μM) of unconjugated PLGA. In parallel, aggregation kinetic of 10 μM Aβ_1–42_ was evaluated with 100 nM PCL, 5 µM PEG-PLGA or 25 µM unconjugated PLGA (50:50 and 75:25 resomers) obtained from another source. Aggregation of 10 μM Aβ_42–1_ was also studied in absence and presence of unconjugated PLGA as a control. The aggregation process was monitored by ThT binding assay while the concentration of ThT was maintained at 20 μM throughout the experiment. The fluorescence signal was measured every 15 min for 24 h using a Fluostar omega BMG Labtech (Aylesbury, UK) with excitation at 440 nm and emission at 480 nm. All kinetic experiments were repeated six times with three technical replicates for each sample/experiment and the data are presented as mean ± SEM for each condition. Raw data for the experiments were normalized in the form of percentage fluorescence intensity and the graphs were plotted using ORIGIN 2018. The kinetic rate of Aβ fibrillization was determined by fitting the fluorescence intensity versus time according to the Boltzmann curve as described below:

$$\textrm{Y} = \frac{{\textrm{F}_{\max } - \textrm{F}_{0} }}{{1 + \textrm{e}^{{(\textrm{T} - \textrm{T}_{\textrm{m}} ) \times \textrm{K}}} }} + \textrm{F}_{\max }$$

where “F_max_” is the maximum fluorescence intensity and “F_0_” is the initial fluorescence intensity, “K” is the rate constant of fibril elongation and “T_m_” is the time at which fluorescence intensity is at half maximum.

### Fluorescence microscopy

A small aliquot (10 μl) of Aβ_1–42_ and Aβ_42–1_ sample in absence and presence of different concentrations of PLGA was added separately on a clean glass slide, air-dried and then stained with ThT solution as described earlier [45]. The images of ThT-stained Aβ_1–42_ and Aβ_42–1_ aggregates were then captured using a fluorescence microscope (Nikon eclipse 90i) at 20X magnification. In parallel, the fluorescence intensity of the ThT-stained Aβ_1–42_ aggregates in the presence and absence of PLGA at saturation was measured using Image J software.

### Scanning transmission electron microscopy (STEM)

An ultra-high resolution Hitachi S-5500 cold field emission STEM was used to examine the morphological changes of Aβ_1–42_ during aggregation. Initially 5 µl Aβ_1–42_ samples with or without PLGA were deposited onto plasma cleaned, carbon coated copper grids for 30 s, excess liquid was blotted and the grids were dried and then gently washed with 10 µl of Milli-Q water to remove the salt from the PBS solution. The grid was stained with 5 µl of 2% aqueous uranyl acetate for 30 s, blotted to remove excess liquid, dried and then imaged at 30 kV accelerating voltage and 30 µA emission current.

### Atomic force microscopy (AFM)

AFM analysis of Aβ samples was performed using Veeco NanoScope IV multimode as described earlier [46]. In brief, 5 μL Aβ samples with or without 25 µM PLGA treatment were deposited on a freshly cleaved mica sheet (1 × 1 cm^2^) by spin-coating at 2500 rpm for 30 s to remove the excessive precipitation from the surface of the sample. The sample surface was then gently washed with 0.5 ml of Milli-Q water under a sterile, dust free environment to remove the excess molecules and the salt from the PBS solution and finally air dried for 1 day before use. The AFM topography images were captured in tapping mode. To obtain an optimized height profile, silicon cantilevers (MikroMasch USA, Inc.) with low spring constants of 4.5 N/m were used in tapping mode (TM-AFM). To obtain a clear image from the surface, a low scan rate (0.5–1 Hz) and amplitude setpoint (1 V) were chosen during measurement.

### Dynamic light scattering (DLS)

The size distribution of aggregated Aβ_1–42_ samples in the absence or presence of PLGA, PEG-PLGA and PCL, at the end of the lag phase and/or after reaching saturation (i.e., 24 h), were carried out with a Malvern Zetasizer-Nano Instrument as described earlier [45]. A He–Ne laser with a wavelength of 632 nm was used to detect backscattered light at a fixed angle of 173°. The aggregated samples were prepared by incubating 10 μM Aβ_1–42_ with or without 5 μM, 10 μM or 25 μM PLGA at 27 °C, 37 °C and 40 °C under constant shaking. The software (DTS v6.20) provides both the mean size and polydispersity by cumulants analysis. The solution viscosity and refractive index (1.33) were assumed to be of water for calculation purposes. Data were collected using a 10 mm quartz cuvette filled with 150 μL sample without agitation from a minimum number of 10 consecutive runs of 10 s each to obtain the autocorrelation function. Particle size was calculated by the manufacturer’s software through the Stokes–Einstein equation assuming spherical particles. In parallel, we used DLS to measure native PLGA’s stability as a function of time at different temperatures (i.e., 27 °C, 37 °C and 40 °C) as well as in medium used in culturing neurons.

### Circular dichroism (CD)

CD experiments were carried out using a Chirascan circular dichroism spectrometer (Applied photophysics) as described earlier [43]. The CD spectra of_._ aggregated Aβ_1–42_ in the absence or presence of 25 µM PLGA (at 1:2.5 ratio) at different temperatures were recorded over a wavelength range of 250–190 nm, by using 0.1 cm path length quartz cell and each spectrum was averaged using 6 repeat scans. The CD spectra of all samples were measured after reaching saturation (i.e., 24 h) to evaluate the effect of PLGA on the aggregation process. Baseline correction was performed by subtracting the spectral contribution of a reaction buffer containing 10 mM Na_2_HPO_4_ and 100 mM NaCl. To retrieve better quantitative structural information, all raw CD spectra were de-convoluted using the CDPro software.

### Isothermal titration calorimetry (ITC)

The thermodynamics of Aβ_1–42_ and PLGA were studied using a Microcal VP-ITC (Malvern) at 298 K as described earlier [47]. In brief, 900 μM PLGA was injected into the sample cell containing 30 μM Aβ_1–42_. All samples were dissolved in PBS and were degassed at 4 °C before titration to prevent the possible formation of air bubbles. To correct for the heat of dilution, standard experiments were performed for Aβ_1–42_ in the absence or presence of PLGA, PLGA with PBS and PBS alone. The volume of first injection was 0.4 μl and the subsequent injections were 2 μl with 120 s spacing. The reference power was set to 10 μcal/sec in high feedback gain mode and the syringe stirring speed was set to 300 rpm. All data were analyzed and fitted by using one binding site in the inbuilt ORIGIN software.

#### Interaction of different Aβ conformers with PLGA

Oligomeric and fibrillar Aβ_1–42_ conformers were first prepared as described earlier [48–50], incubated with 25 µM PLGA for 24 h and then processed for the filter-trap analysis using conformer specific Aβ antibodies. In brief, 10 µl Aβ_1–42_ samples with or without PLGA were spotted on a nitrocellulose membrane (0.02 µm), subjected to vacuum filtration through a 96-well Bio-Dot Microfiltration system, washed with Tris-buffered saline and then incubated at 4 °C for 12 h with oligomer specific A11 as well as aggregate specific OC Aβ antibodies (Table 1). The membranes were further washed with buffers, incubated with appropriate horseradish peroxidase-conjugated secondary antibodies (1:5000) and developed with an ECL kit. All blots were examined using a FluorChem E system (Santa Clara, CA, USA) and the images were processed using Image J software.

### Mouse cortical neuronal cultures and cell viability

Timed pregnant BALB/c mice purchased from Charles River (St. Constant, Quebec, Canada) were maintained according to Institutional and Canadian Council on Animal Care guidelines. Primary cortical cultures were prepared from 18-day-old embryos of timed pregnant mice as described previously [51, 52]. In brief, the frontal cortex from pup brains was dissected in Hanks’ balanced salt solution supplemented with 15 mM HEPES, 10 units/ml penicillin and 10 µg/ml streptomycin and then digested with 0.25% trypsin–EDTA. The cell suspension was filtered through a cell strainer and plated (1.5 × 10^4^ cells/cm^2^) on 96-well plates. The cultures were grown at 37 °C in a 5% CO_2_ humidified atmosphere in Neurobasal medium supplemented with B27/N2, 50 μM L-glutamine, 15 mM HEPES, 10 units/ml penicillin, 10 mg/ml streptomycin and 1% FBS. The medium was replaced 1 day later without FBS and neurons are treated on day 6 after plating. In brief, cultured neurons were first treated with oligomeric 1-20 μM Aβ_1–42_ for 24 h. In a parallel set of experiment, cultured neurons were co-treated with unconjugated PLGA (1–25 µM), PEG-PLGA (5 µM) or PCL (100 nM) along with 10 μM oligomeric Aβ_1–42_ for 24 h. Additionally, cultured neurons were treated with 10 µM Aβ_1–42_ aggregates incubated with or without 25 µM unconjugated PLGA for 24 h or 72 h at 37 °C. The control and treated cultured neurons after 24 h were processed for cell viability/toxicity assays as well as for Western blotting.

### ***Administration of PLGA with or without Aβ***_***1***–***42***_*** into control mouse brains***

To determine if native PLGA can influence the Aβ_1–42_ clearance from the brain, we used normal control mice on C57BL/6 J background. These mice were purchased from The Jackson Laboratory (Bar Harbor, ME, USA) and housed on a 12 h light/dark cycle with access to food and water ad libitum in accordance with Canadian Council on Animal Care guidelines. Six-month-old control mice (n  = 3/group) were stereotaxically (− 0.2 mm mid/lateral, − 1.6 mm antero/posterior and − 1.2 mm dorso/ventral from Bregma) injected using Hamilton syringe under anesthesia with either 5 µl fluorescence labelled Aβ_1–42_ (10 µM) followed by CSF or 5 µl native PLGA (25 µM) [53]. The mice were decapitated after 6 and 24 h post-injection, their brains were snap-frozen and sectioned (35 µm) using a cryostat. The brain sections were then mounted with ProLong™ gold antifade reagent with DAPI and imaged using a Nikon Eclipse 90i fluorescence microscope equipped with a Retiga 2000R Q imaging system (Nikon Instruments Inc., NY, USA). The fluorescence intensity of labelled Aβ and DAPI was quantified from the acquired photomicrographs using image-J software and the relative fluorescence intensity of Aβ was calculated as the ratio of Aβ *vs* corresponding DAPI intensities [54].

### Cytotoxicity assays

The viability of control and treated neurons following various experimental paradigms was determined using the MTT and/or LDH assays as previously described [44, 45]. For the MTT assay, the culture media of the neurons was replaced with new media containing 0.5 mg/ml MTT, the cells were incubated for 4 h at 37 °C, the formazan crystals were dissolved in dimethyl sulfoxide and absorbance was measured at 570 nm with a microplate reader. To substantiate the MTT data, the viability of control and treated cultured neurons was measured using a commercial LDH activity assay kit. The absorbance for LDH was measured at 490 nm with a Spectramax M5 spectrophotometer. All cell viability/toxicity experiments were repeated three times with three to five technical replicates per sample.

### Western blotting

Western blotting was performed on control and Aβ-treated cultured cells as described earlier [42]. In brief, cultured neurons from various experimental paradigms were homogenized with radioimmunoprecipitation lysis buffer and total protein content was quantified using a BCA kit. Denatured samples were resolved on 10% polyacrylamide gels, transferred to PVDF membranes, blocked with 5% milk and incubated overnight at 4 °C with various primary antibodies at dilutions listed in Table 1. The membranes were then incubated with horseradish peroxidase-conjugated secondary antibodies (1:5000) and immunoreactive proteins were detected with an ECL kit. All blots were re-probed with anti-β-actin antibody and quantified using ImageJ as described earlier [55].

### Statistical analysis

All data collected from a minimum of 3 biological repeats with each experiment performed in at least three replicates were expressed as means  ±  SEM. The cell viability data from cultured neurons were analyzed by one-way ANOVA followed by Bonferroni’s *post-hoc* analysis for multiple comparisons with a significance threshold set at *p * < 0.05. All statistical analysis was performed using GraphPad Prism (GraphPad Software, Inc., CA, USA).

## Results

### ***Effect of temperatures on Aβ***_***1***–***42***_*** aggregation***

**Fig. 1:**
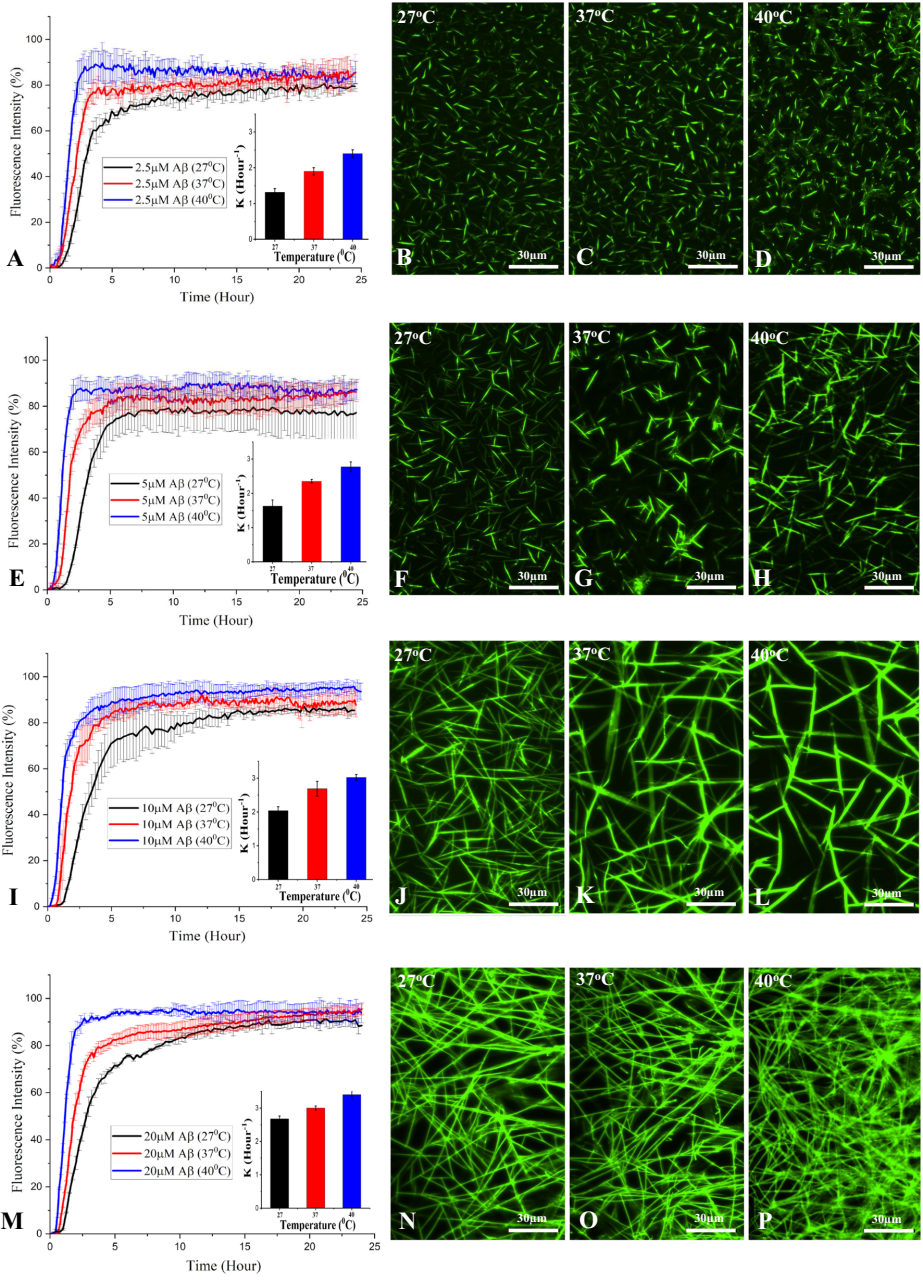
Aggregation of Aβ_1–42_ at different temperatures. ThT kinetic assays showing the spontaneous aggregation curves and the corresponding fluorescence images of 2.5 µM (**A**–**D**), 5 µM (**E**–**H**), 10 µM (**I**–**L**) and 20 µM (**M**–**P**) Aβ_1–42_ over 24 h incubation at 27 °C (black), 37 °C (red) and 40 °C (blue). Note the aggregation of Aβ_1–42_ increases as functions of dose and temperature which reach plateaus over time as indicated by ThT fluorescence levels. The fluorescence images of different Aβ_1–42_ concentrations were taken following 24 h incubation at 27 °C, 37 °C and 40 °C. Rate constant of the kinetic reaction “K” at different concentrations of Aβ_1–42_ are shown in the inset of respective aggregation kinteic curves

Aggregation of Aβ peptide plays an important role in AD pathogenesis. Structurally, Aβ fibrils are aggregates with repetitive cross-β sheets stabilized by different molecular interactions. The classic dye ThT is normally used in detecting Aβ fibril formation due to its strong fluorescence emission upon binding to cross-β fibril structures [43, 44]. Since temperature is known to influence protein aggregation which underlies the development of AD pathology [17–21], it is of clinical relevance to determine how variation in temperatures can affect spontaneous Aβ_1–42_ aggregation. Thus, we performed ThT kinetic assay by incubating 2.5–20 µM human Aβ_1–42_ at three different temperatures (27, 37 and 40 °C) over 24 h as described earlier (Fig. 1A–P). The aggregation kinetics in all studied conditions exhibited sigmoidal curves with a lag phase and a log phase followed by a saturation phase (Fig. 1A, E, I, M). With increasing concentrations of Aβ_1–42_ from 2.5 to 20 µM at a particular temperature, the rate and amount of fibril formation increases as is evident from the corresponding ThT fluorescence values at the saturation level (Fig. 1, Additional file 1: Fig. S1). These results reflect the general law of aggregation where an increase in the concentration of an amyloidogenic protein is known to enhance its aggregation as a function of time. Similarly, the aggregation rate of Aβ_1–42_ at a given concentration also increases with a rise in temperature from 27 to 40 °C (insets in Fig. 1A, E, I, M). This is simply because time taken to form fibrils decreases with a rise in temperature due to an increase in the rate of thermal effective collisions. This is apparent more at 5, 10 and 20 µM than 2.5 µM Aβ_1–42_ over a 24 h incubation period. Interestingly, unlike concentrations, the rise in temperature displays marginal changes in the ThT fluorescence value at saturation indicating no drastic alterations in the formation of total fibrils for a given Aβ_1–42_ concentration at different temperatures. The propensity of Aβ aggregation at saturation was further validated by fluorescence imaging of ThT labelled samples which showed a dose-dependent increase of fibrillar Aβ entities at a given temperature. Formation of fibrils also increases as a function of temperature for a given concentration of Aβ_1–42_ (Fig. 1B–D, F–H, J–L, N–P). In contrast to normal Aβ_1–42_, 10 µM Aβ_42–1_ (i.e., a negative control) did not aggregate either at 27, 37 or 40 °C during 24 h incubation period (Additional file 1: Fig. S2).

### ***Effects of PLGA on temperature-dependent spontaneous Aβ***_***1***–***42***_*** aggregation***

**Fig. 2:**
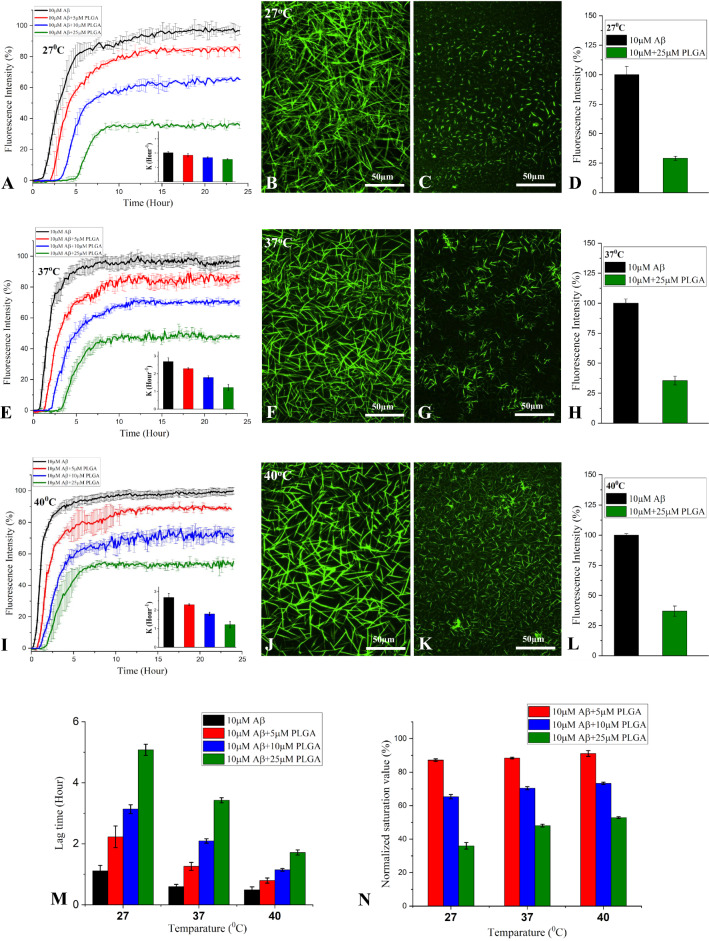
Attenuation of Aβ_1–42_ aggregation by PLGA at different temperatures. PLGA dose-dependently (5–25 µM) attenuates spontaneous aggregation of 10 µM Aβ_1–42_ as revealed by ThT kinetic assays and respective fluorescence images/quantification over 24 h incubation at 27 °C (**A**–**D**), 37 °C (**E**–**H**) and 40 °C (**I**–**L**). The corrosponding rate constant of the kinetic reaction “K” is shown as inset in each plot. ThT stained fluorescence images (**B**, **C**, **F**, **G**, **J**, **K**) and the corresponding quantification (**D**, **H**, **L**) of PLGA untreated and treated Aβ_1–42_ samples were taken after 24 h incubation at 27 °C, 37 °C and 40 °C. Histograms representing the attenuated fluorescence values at lag-time (**M**) and at saturation (**N**) of Aβ_1–42_ kinetic reaction in the absence and presence of 5-25 µM PLGA at different temperatures

Prior to determining the effects of unconjugated PLGA on temperature-dependent Aβ aggregation, we showed that PLGA nanoparticles, as observed in STEM and AFM, displayed spheroidal morphology. Our DLS analysis further revealed that PLGA with diameter  ~ 100 nm is quite stable over 48 h period at 27, 37 and 40 °C (Additional file 1: Fig. S3A–F). The effects of 5–25 µM unconjugated PLGA on aggregation kinetics of 10 µM Aβ_1–42_ at 27, 37 and 40 °C were subsequently evaluated over a 24 h period using the ThT assay. Our data clearly show that PLGA dose-dependently inhibited spontaneous Aβ_1–42_ aggregation at all temperatures (Fig. 2A–N). At the low concentration of 5 µM (i.e., PLGA:Aβ  = 1:2), PLGA displayed only a slight inhibitory effect on Aβ aggregation at different temperatures, whereas 10 µM PLGA (i.e., PLGA:Aβ  = 1:1) noticeably attenuated Aβ aggregation at various temperatures relative to that of pure Aβ. When the PLGA concentration was increased to 25 µM (i.e., PLGA:Aβ  = 2.5:1) a much stronger inhibition was observed at all temperatures, with a reduction of  ~ 65% at 27 °C,  ~ 52% at 37 °C and  ~ 48% at 40 °C in the saturation phase over the 24 h incubation (Fig. 2A, E, I, N). Increasing concentrations of PLGA were found to prolong the lag time and decrease the rate of Aβ fibrilization at all temperatures, but this effect is more pronounced at 27 °C than at higher temperatures (Fig. 2A, E, I, M). Suppression of Aβ_1–42_ aggregation at saturation by 5 or 10 µM PLGA, as apparent from ThT kinetic assays, did not display striking effects as a function of temperature. However, 25 µM PLGA inhibited Aβ aggregation more profoundly at 27 °C than at 37 or 40 °C over a 24 h incubation period (Fig. 2N). The inhibitory effect of PLGA on spontaneous Aβ_1–42_ aggregation at saturation is further validated by fluorescence imaging which showed respectively fewer Aβ fibrillar entities in the presence of PLGA (Fig. 2B–D, F–H, J–L) with a corresponding decrease in temperatures from 40 to 27 °C (i.e., 37% at 40 °C, 35% at 37 °C and 29% at 27 °C).

**Fig. 3:**
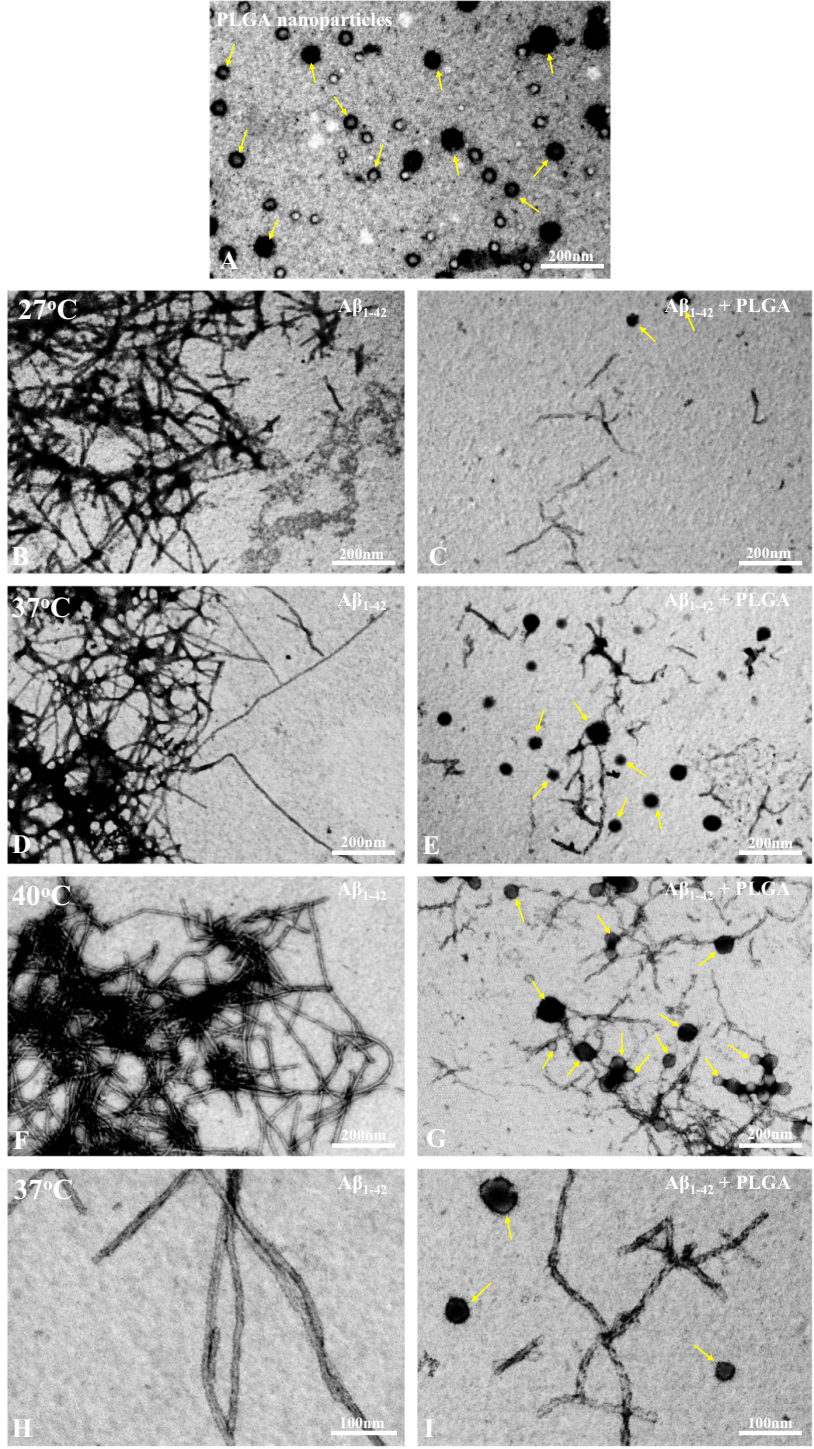
Attenuation of Aβ_1–42_ aggregation by PLGA as revealed by STEM. **A** STEM image showing the spherical morphology of unconjugated PLGA nanoparticles without Aβ peptide. STEM images of 10 µM Aβ_1–42_ following 24 h incubation in the absence (**B**, **D**, **F**) and presence (**C**, **E**, **G**) of 25 µM PLGA at 27 °C (**B**, **C**), 37 °C (**D**, **E**) and 40 °C (**F**, **G**). Note the relative decrease in the amount of Aβ fibrils at different temperatures in presence of 25 µM PLGA. STEM images showing higher magnification of Aβ_1–42_ samples in the absence (**H**) and presence (**I**) of 25 µM PLGA at 37 °C. Note the localization of twisted fibers in the PLGA-treated Aβ sample. PLGA nanoparticles are indicated with arrows

**Fig. 4:**
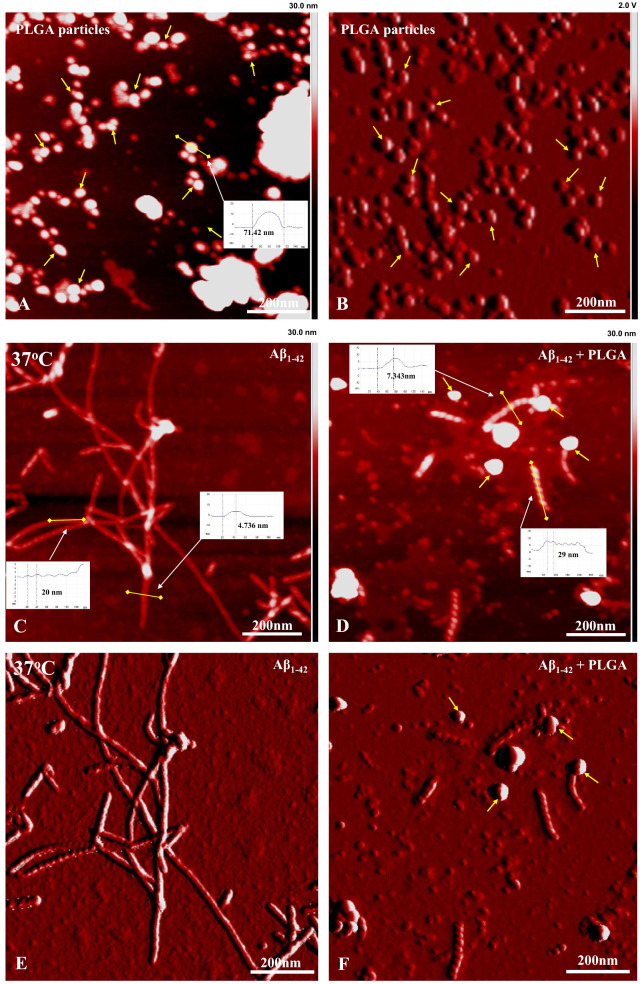
Attenuation of Aβ_1–42_ aggregation by PLGA as revealed by AFM. AFM images in height (**A**) and amplitude (**B**) modes showing spherical morphology of unconjugated PLGA nanoparticles without Aβ peptide. AFM images in height (**C**, **D**) and amplitude (**E**, **F**) modes of 10 µM Aβ_1–42_ following 24 h incubation in the absence (**C**, **E**) and presence (**D**, **F**) of 25 µM PLGA at 37 °C. Note the formation and structural details of different populations of smaller aggregates such as short twist fibrils and oligomers in presence of 25 µM PLGA compared to untreated control Aβ_1–42_. PLGA nanoparticles are highlighed with arrows

To delve into the structural details of Aβ aggregates in the presence or absence of 25 µM PLGA after 24 h incubation, we used STEM (Fig. 3A–I) and AFM (Fig. 4A–F) to examine aggregate morphology at higher resolution. Our data revealed that PLGA nanoparticles, which displayed spheroidal morphology with an average diameter of  ~ 100 nm (Figs. 3A;  4A, B), are associated directly with Aβ_1–42_ fibers and attenuate peptide aggregation, leading to the formation of a heterogenous mixture of smaller Aβ aggregates (Figs. 3B–I;  4C–F). The population of Aβ fibers/aggregates, as revealed by our STEM images, decreases at all studied temperatures but is more evident at lower temperature than at higher temperature (Fig. 3B–I). At the structural level, Aβ fibrils after 24 h incubation without PLGA appear mostly as aggregated long straight and twisted fibers which increase in number with the rise of temperature. Conversely, the PLGA treated Aβ sample shows short, twisted fibers as well as small globular spherical aggregates without any filamentous structure (most likely Aβ oligomers) (Fig. 3C, E, G, I). With the rise of temperature from 27 to 40 °C, the population of twisted fibers increases with a concomitant decrease in small globular aggregates (Fig. 3C, E, G, I)—suggesting a possible shift/formation of more twisted fibers. Our AFM analysis, apart from two populations (Fig. 4C–F), reveals additional structural differences of Aβ aggregates in the absence and presence of PLGA in both height (Fig. 4C, D) and amplitude (Fig. 4E, F) modes. The untreated control Aβ fibers showed a helical pitch of  ~ 20 nm and a height of  ~ 4.7 nm from the surface, whereas the PLGA-treated samples show short, twisted fibrils with a  ~ 29 nm helical pitch and  ~ 7.3 nm in height from the surface (Fig. 4C–F). This alteration raises the possibility that PLGA may trigger loosening of molecular packing and intermolecular interaction between intertwined protofilaments/fibrils leading to the formation of short fibrillar structures.

**Fig. 5:**
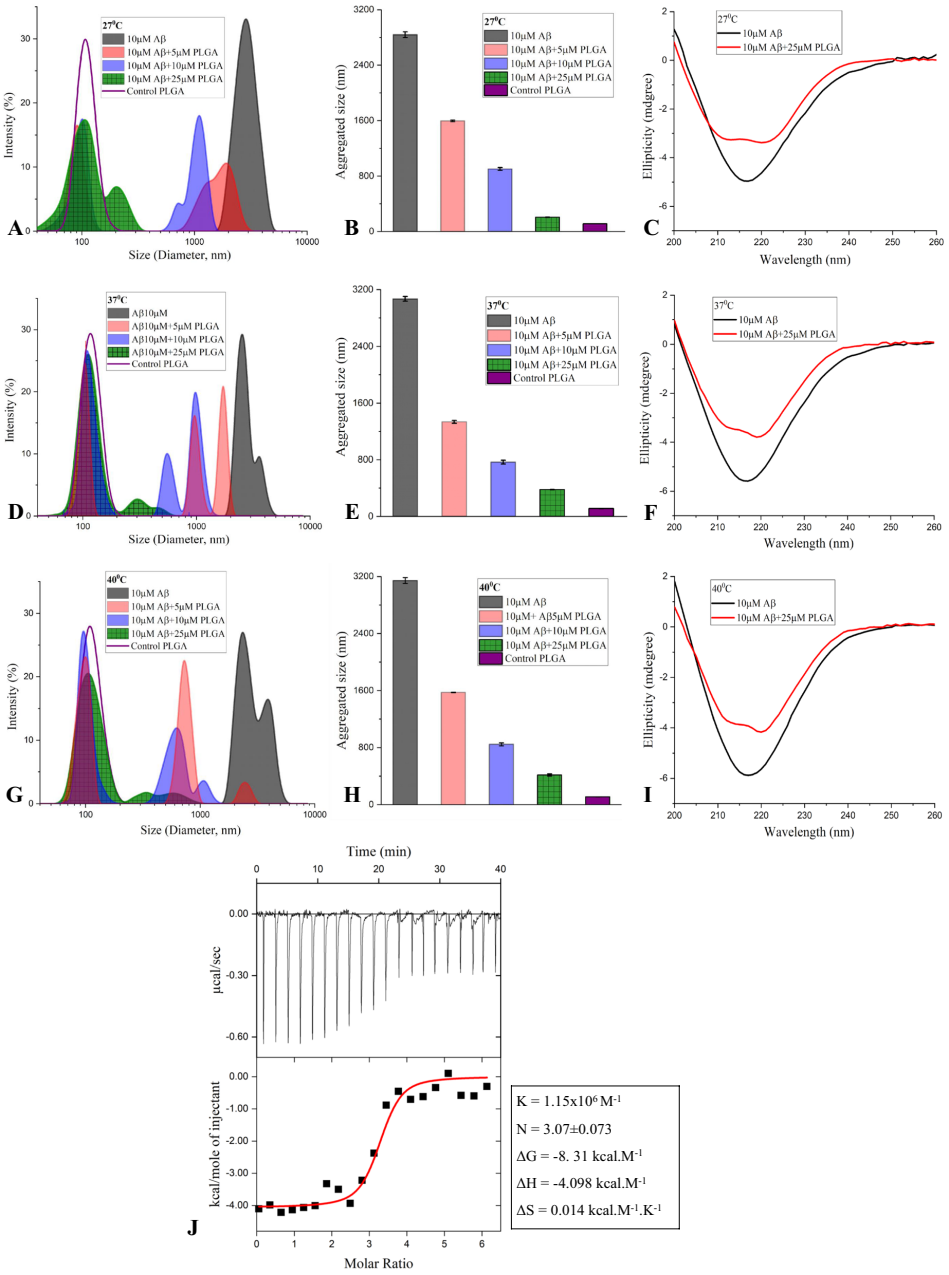
PLGA changes size and secondary structure of Aβ_1–42_ aggregates. DLS analysis (**A**, **B**, **D**, **E**, **G**, **H**) and CD spectra (**C**, **F**, **I**) of 10 µM Aβ_1–42_ in the presence and absence of PLGA following 24 h incubation at 27 °C (**A**–**C**), 37 °C (**D**–**F**) and 40 °C (**G**–**I**). DLS analysis reveals a differential decrease in the diameter of Aβ_1–42_ in the presence of 5 µM (red), 10 µM (blue) and 25 µM (green) PLGA incubation at 27 °C (**A**, **B**), 37 °C (**D**, **E**) and 40 °C (**G**, **H**) compared to control Aβ_1–42_ (black) as depicted by size distribution curves and avearge size histograms. PLGA nanoparticles (violet) without exposure to Aβ peptide have also been represented in the corresponding graphs. CD spectra showing β-sheet content following incubation of 10 µM Aβ_1–42_ in the absence (black) and presence (red) of 25 µM PLGA after 24 h incubation at 27 (**C**), 37 (**F**) and 40 °C (**I**), respectively. Note the decreased formation of β-sheet rich secondary in the presence of PLGA which increases with the rise of temperature. (**J)** ITC binding isotherms showing interaction between PLGA and Aβ_1–42_ at 25 °C. The top panel shows the differential power signal measured for each injection throughout the experiment and the bottom panel shows the integrated peak areas corresponding to the measured heat released per injection. The thermodynamic parameters for the protein and ligand interaction are shown in the adjacent table

To further validate an overall reduction in the fibrillar size/entities following PLGA treatment, we performed DLS analysis of Aβ samples with or without PLGA following aggregation kinetic assays. Our results indeed showed a reduction in hydrodynamic radii of Aβ aggregates with multiple peaks at all temperatures as a function of PLGA concentrations, but the effect was more profound at 27 °C than at 37 or 40 °C over 24 h incubation (Fig. 5A, B, D, E, G, H). To better understand the internal architecture which transforms the long fibrils to shorter species, we performed CD spectroscopy of Aβ samples with or without PLGA treatment after 24 h incubation. Consistent with earlier results [43], the far-UV CD spectra of Aβ_1–42_ fibrils after 24 h at different temperatures showed characteristic β-sheet conformation with minima around 216 nm (Fig. 5C, F, I), suggesting the presence of β-sheet rich secondary structures. Although the nature of the secondary structure of Aβ_1–42_ fibrils remains same at all temperatures, the β-sheet content, as revealed by deconvolution of CD spectra, increases with temperature in monotonic order of efficacy i.e., 40 °C  > 37 °C  > 27 °C (Table 2). In contrast to Aβ_1–42_ alone, the presence of PLGA enhanced the α-helical and decreased β-sheet contents at all temperatures (Fig. 5C, F, I). The reciprocity of α-helicity vs β-sheet content correlates positively as a function of temperature increase from 27 to 40 °C as shown in Table 2. Our ITC experiments involving titration of native PLGA to a solution containing Aβ_1–42_ showed an exothermic isotherm profile with a binding constant, K  = 1.15 × 10^6^ M^−1^ and a stoichiometry of  ~ 3 (N  = 3.07). The Gibbs free energy ΔG was found to be − 8. 31 kcal.M^−1^ with an Enthalpy (ΔH)  = − 4.098 kcal.M^−1^ and an Entropy (ΔS)  = 0.014 kcal.M^−1^.K^−1^ (Fig. 5J).

**Table 2:** CD spectroscopy data depicting α-helical and β-sheet contents following 24 h aggregation of 10 µM Aβ_1–42_ at different temperatures in the absence and presence of 25 µM unconjugated PLGA

	α-helical content (%)	β-sheet content (%)
Aβ_1–42_ (27 °C)	2	39
Aβ_1–42_ (37 °C)	2	42
Aβ_1–42_ (40 °C)	2.1	46
Aβ_1–42_ + PLGA (27 °C)	24	32
Aβ_1–42_ + PLGA (37 °C)	20	36
Aβ_1–42_ + PLGA (40 °C)	18	39

**Fig. 6:**
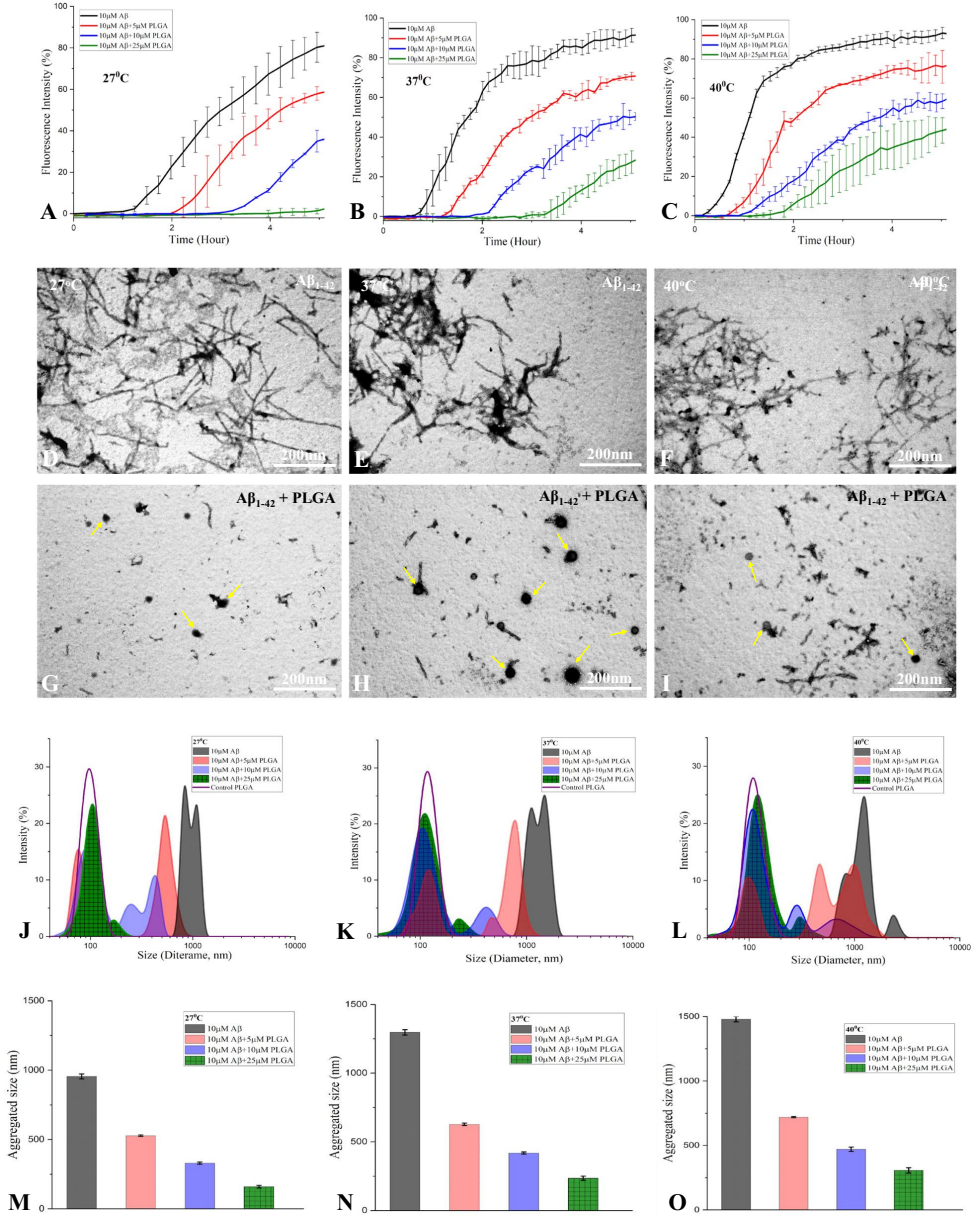
PLGA alters the lag phase of Aβ_1–42_ aggregation kinetics at different temperatures. ThT kinetic assay showing aggregation of 10 µM Aβ_1–42_ in the absence and presence of 5–25 µM of PLGA over 5 h incubation (lag phase) at 27 °C (**A**), 37 °C (**B**) and 40 °C (**C**). Note the decreased duration of the lag phase with rise in temperature in the absence and presence of presence of PLGA. STEM images of 10 µM Aβ_1–42_ in the absence (**D**–**F**) and presence of 25 µM PLGA (**G**–**I**) after 5 h incubation at 27 °C (**D**, **G**), 37 °C (**E**, **H**) and 40 °C (**F**, **I**). Note the attenuation of Aβ fibril formation in the presence of PLGA nanoparticles. DLS analysis revealing diameter (**J**–**L**) and average size (**M**–**O**) of 10 µM Aβ_1–42_ in the absence and presence of 25 µM PLGA after 5 h incubation at 27 °C (**J**, **M**), 37 °C (**K**, **N**) and 40 °C (**L**, **O**). Note the attenuation of Aβ aggregation at different temperatures as a function of PLGA concentrations. PLGA nanoparticles are highlighed with arrows

The inhibitory effects of PLGA on spontaneous Aβ_1–42_ aggregation appear to be mediated by a decreased fibrilization rate and increased lag time which ensue as a function of temperature in the following order of efficacy i.e., 27 °C  > 37 °C  > 40 °C (Fig. 6A–C). This is supported by a decreased population of Aβ fibers/aggregates observed in the presence of 25 µM PLGA at 27 °C than at higher temperatures using STEM (Fig. 6D–I). Our DLS analysis also revealed a mean size reduction in the hydrodynamic radii of Aβ aggregates in the presence of increasing concentrations of PLGA during the lag phase of Aβ aggregation (Fig. 6J–O). The decreased population of Aβ fibers/aggregates is though evident at all temperatures in the presence of PLGA, it is predominant at 27 °C than at higher temperatures (i.e., 37 or 40 °C) (Fig. 6M–O).

### ***Effects of different PLGA resomer, PEG-PLGA and PCL on temperature-dependent spontaneous Aβ***_***1***–***42***_*** aggregation***

**Fig. 7:**
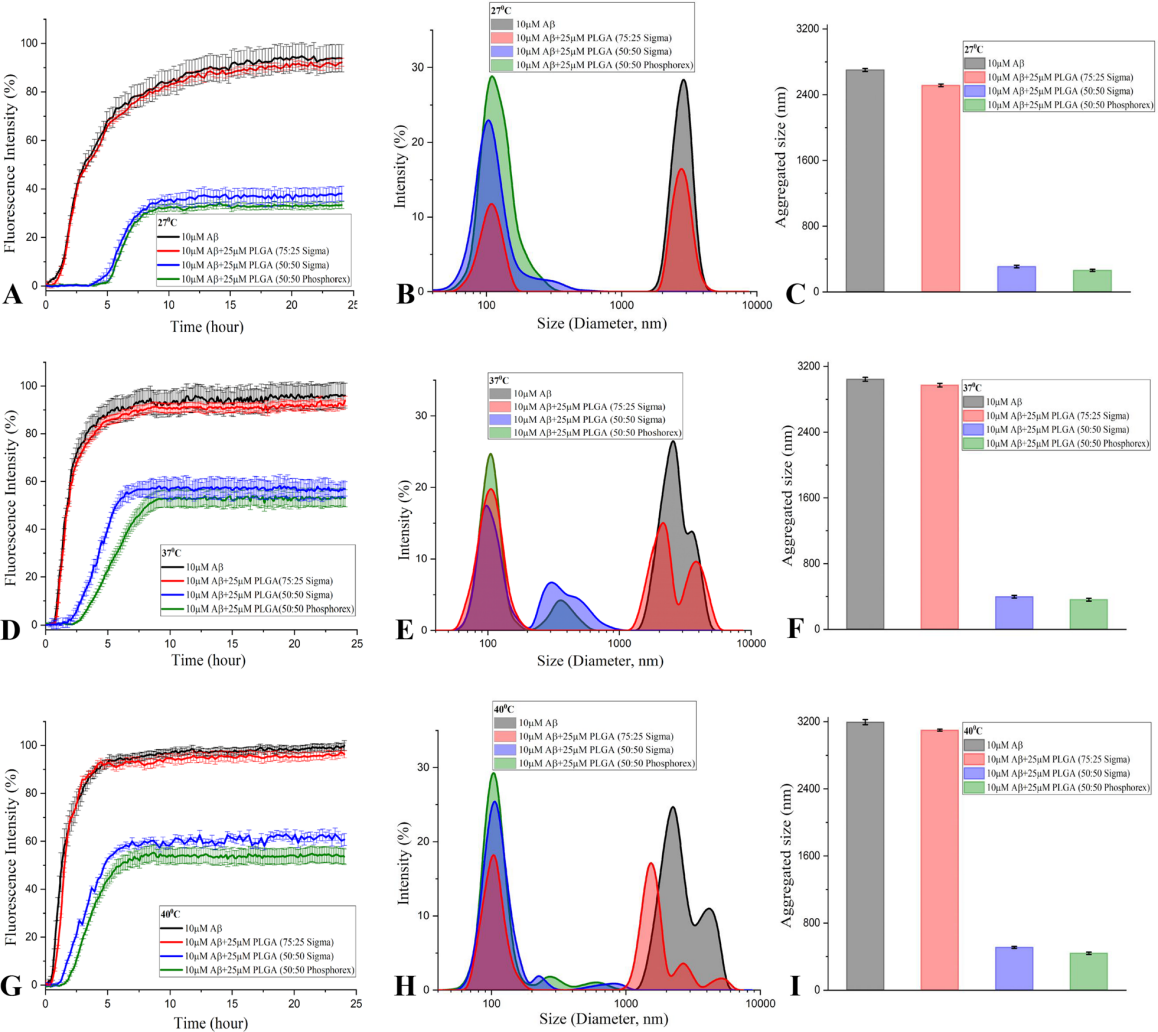
Resomer specific attenuation of spontaneous Aβ_1–42_ aggregation by PLGA. ThT kinetic assays (**A**, **D**, **G**) and DLS analysis (**B**, **C**, **E**, **F**, **H**, **I**) showing that 50:50 resomer PLGA from Phosphorex (green) and Sigma (blue), but not 75:25 resomer PLGA (red), was able to suppress spontaneous aggregation of 10 µM Aβ_1–42_ over 24 h incubation at 27 °C (**A**–**C**), 37 °C (**D**–**F**) and 40 °C (**G**–**I**). DLS analysis revealing diameter and average size histograms of Aβ_1–42_ in the presence of 25 µM PLGA of different resomers at 27 °C (**B**, **C**), 37 °C (**E**, **F**) and 40 °C (**H**, **I**) compared to control Aβ_1–42_ (black). Note the attenuation of Aβ aggregation by 50:50 resomer PLGA but not with 75:25 PLGA resomer compared to control Aβ_1–42_

**Fig. 8:**
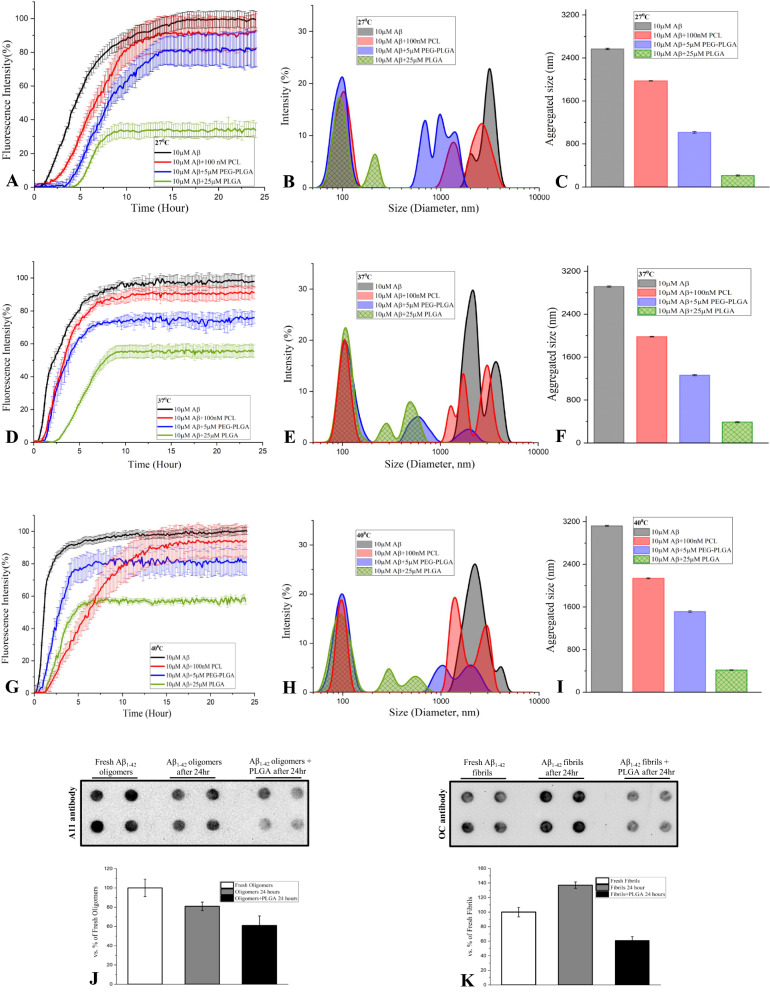
Attenuation of spontaneous Aβ_1–42_ aggregation by PLGA vs PCL and PEG-PLGA. ThT kinetic assays (**A**, **D**, **G**) and DLS analysis (**B**, **C**, **E**, **F**, **H**, **I**) showing effects of PLGA (25 µM; green) compared to PEG-PLGA (5 µM; blue) and PCL (100 nM; red) on spontaneous aggregation of 10 µM Aβ_1–42_ (black) over 24 h incubation at 27 °C (**A**–**C**), 37 °C (**D**–**F**) and 40 °C (**G**–**I**). DLS analysis revealing diameter and average size histograms of Aβ_1–42_ in the presence of PLGA (green), PEG-PLGA (blue) and PCL (red) at 27 °C (**B**, **C**), 37 °C (**E**, **F**) and 40 °C (**H**, **I**) compared to control Aβ_1–42_ (black). Note that PLGA is more potent in suppressing spontaneous Aβ_1–42_ aggregation than PEG-PLGA and PCL at the studied concentrations. Filter-trap assay and respective quantification data revealing specificty of oligomeric and fibrillar Aβ_1–42_ conformers and their interactions in the presence or absence of 25 µM PLGA nanoparticles as detected using oligomeric specific A11 antobody (**J**) and fibrillar specific OC antibody (**K**). Note that presence of PLGA attenauted the detection of both Aβ_1–42_ conformers but the interaction is somewhat more evident with Aβ aggregates than with Aβ oligomers

To determine if PLGA with 50:50 resomer composition from another source can suppress Aβ_1–42_ aggregation at different temperatures (27, 37 and 40 °C), we performed ThT kinetic assays as well as DLS analysis using 10 µM Aβ_1–42_ in the presence or absence of 25 µM PLGA obtained from Sigma-Aldrich (Fig. 7A–I). Our data clearly revealed that 50:50 resomer PLGA from Sigma, as observed with Phosphorex, can suppress aggregation of Aβ peptides at all temperatures but the effect is more profound at 27 °C than at 37 or 40 °C over a 24 h incubation (Fig. 7A, D, G). This is further validated by DLS analysis which showed a reduction in diameter of Aβ aggregates in presence of 25 µM PLGA in the following order of efficacy i.e., 27 °C  > 37 °C  > 40 °C (Fig. 7B, C, E, F, H, I). However, equimolar PLGA with 75:25 resomer composition did not alter Aβ_1–42_ aggregation as evident by ThT kinetic assay or DLS analysis (Fig. 7A–I). As a follow up to these results, we evaluated if other polymorphic nanoparticles such as PEG-PLGA and PCL can suppress Aβ_1–42_ aggregation [56, 57]. For this experiment we used 5 µM PEG-PLGA and 100 nM PCL as they were found to protect neurons against toxicity following 24 h exposure to 10 µM Aβ_1–42_ (Additional file 1: Fig. S4A, B). Interestingly, both 5 µM PEG-PLGA and 100 nM PCL suppress to some extent spontaneous Aβ_1–42_ aggregation at 27, 37 or 40 °C over a 24 h incubation period but the effect is much less potent than those observed with native PLGA. This is evident both in ThT kinetic assay and DLS analysis (Fig. 8A–I). These data indicate the specificity of the effects mediated by 50:50 resomer of PLGA which is commonly used in various biological experiments.

### Effects of PLGA on different Aβ conformers

To validate the interaction between PLGA and Aβ peptide, oligomeric and fibrillar Aβ_1–42_ were first prepared as described earlier [47–49]. Subsequently, oligomeric and fibrillar Aβ_1–42_ was incubated with 25 µM unconjugated PLGA for 24 h and then probed with conformer-specific A11 and OC Aβ antibodies using filter-trap assay. Our results clearly demonstrate that PLGA interacted with both conformers of Aβ_1–42_, but its binding to Aβ aggregates was somewhat more apparent than Aβ oligomers under the given experimental conditions (Fig. 8J, K).

### ***Effects of PLGA on temperature-dependent Aβ***_***1***–***42***_*** disassembly***

**Fig. 9:**
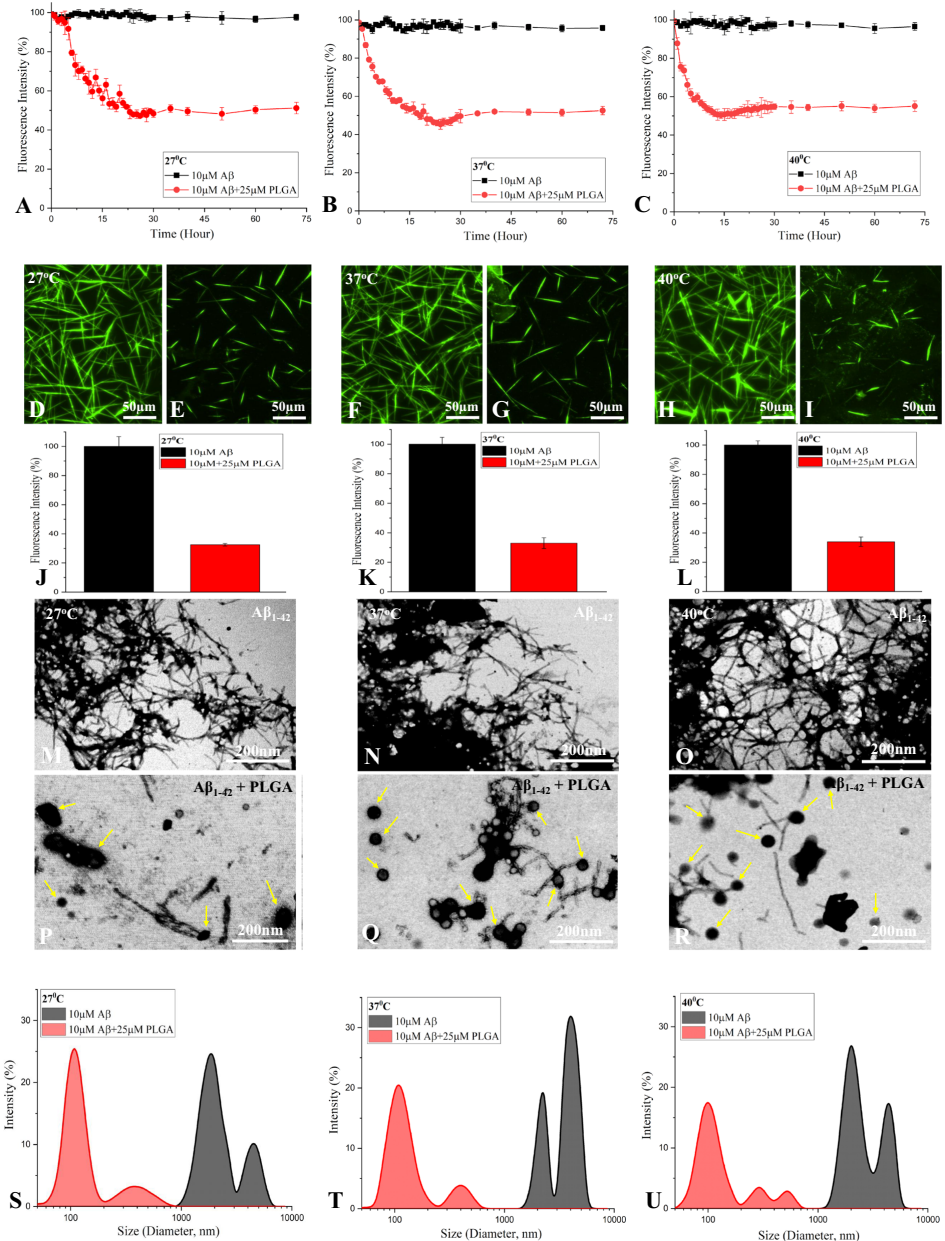
PLGA disassembles preaggregated Aβ_1–42_ fibers. ThT kinetic assays showing the disassembly of mature Aβ_1–42_ fibers and the corresponding fluorescence images in the absence and presence of 25 µM PLGA over 72 h incubation at 27 °C (**A**, **D**, **E**), 37 °C (**B**, **F**, **G**) and 40 °C (**C**, **H**, **I**). Note the faster rate of Aβ disaggegation with the rise of temperature. Histograms showing the quantification of fluorescence intensity measured in the absence and presence of 25 µM PLGA over 72 h incubation at 27 °C (**J**), 37 °C (**K**) and 40 °C (**L**) at saturation which did not differ markedly. STEM images showing matured Aβ_1–42_ fibers in the absence (**M**–**O**) and presence of 25 µM PLGA (**P**–**R**) after 72 h incubation at 27 °C (**M**, **P**), 37 °C (**N**, **Q**) and 40 °C (**O**, **R**). Note the presence of PLGA nanoparticles (arrows) in conjugation with disassembled Aβ fibers at different temperatures. DLS analysis representing diameter of different aggregated Aβ_1–42_ populations in the absence and presence of 25 µM PLGA after 72 h incubation at 27 °C (**S**), 37 °C (**T**) and 40 °C (**U**). Note the decreased diameter of aggregated Aβ_1–42_ populations in the presence of 25 µM PLGA at different temperatures

To establish if PLGA can promote disassembly of mature Aβ fibers at different temperatures, preformed Aβ_1–42_ fibers were incubated with 25 µM PLGA for 72 h at various temperatures (i.e., 27, 37 and 40 °C). Our ThT kinetic assay revealed that matured Aβ_1–42_ fibers can initially be dismantled by PLGA in a time-dependent manner at all temperatures and then remained stable over 72 h incubation period. The dissociation rate is found to be somewhat faster with increasing temperatures, albeit the relative levels of disassembled Aβ fibers at the end of incubation did not exhibit marked variation between temperatures (i.e.,  ~ 52% at 27 °C,  ~ 53% at 37 °C and  ~ 55% at 40 °C) (Fig. 9A–C). This is supported by fluorescence imaging (32% at 27 °C, 33% at 37 °C and 34% at 40 °C, Fig. 9D–L) as well as STEM (Fig. 9M–R) data showing comparable presence of smaller Aβ fragments after 72 h PLGA treatment at all temperatures. Dismantling of Aβ_1–42_ fibers is also apparent by DLS at 27, 37 and 40 °C following 72 h PLGA treatment with a shift towards the lower ordered entities (diameter  ~ 100–600 nm) compared with untreated Aβ_1–42_ fibers (diameter  ~ 1000–8000 nm) (Fig. 9S–U).

### PLGA-mediated attenuation of Aβ aggregation protects cultured neurons

**Fig. 10:**
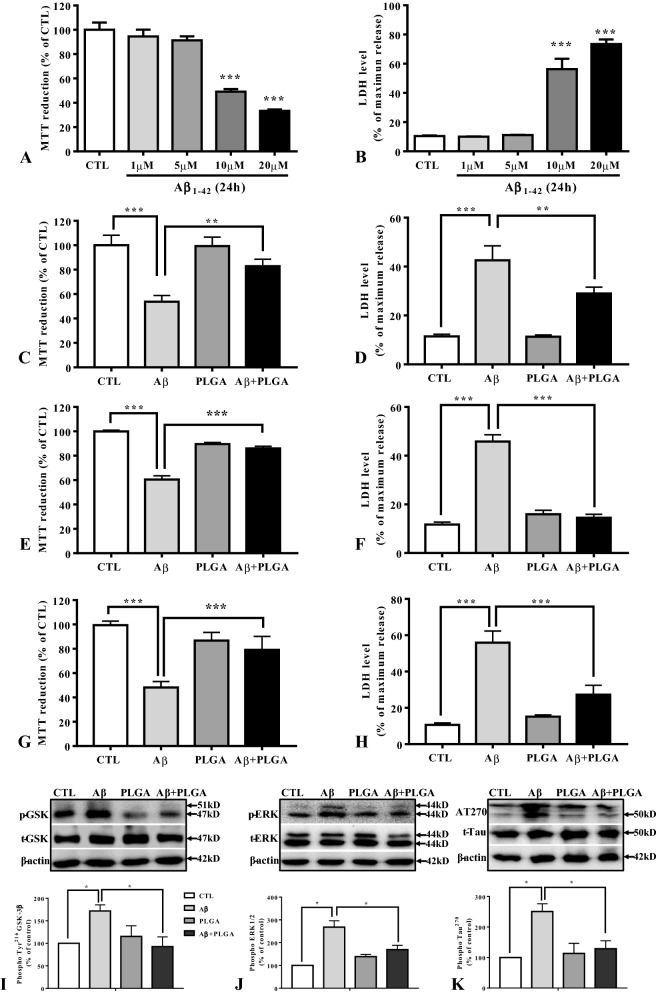
PLGA nanoparticles protect cultured neurons. Histogram depicting dose-dependent decrease in the viability of mouse cortical cultured neurons following 24 h exposure with oligomeric human Aβ_1–42_ compared to control (CTL) neurons as revealed by MTT (**A**) and LDH (**B**) assays. Histograms showing protection of mouse cultured neurons following co-treatment of 10 µM Aβ_1–42_ with 25 µM PLGA over 24 h as detected with MTT (**C**) and LDH (**D**) assays. Histograms showing protection of primary cortical neurons following treatment with Aβ_1–42_ samples collected after 25 µM PLGA-mediated spontaneous attenuation of Aβ_1–42_ aggregation (**E**, **F**) and disassembled of matured Aβ_1–42_ fibers (**G**, **H**) at 37 °C as detected with MTT (**E**, **G**) and LDH (**F**, **H**) assays. Immunoblots and histograms showing that protective effects following attenuation of spontaneous Aβ aggregation by PLGA are associated with a decrease in the levels of Phospho-Tyr^216^ GSK-3β (**I**), Phospho-ERK1/2 (**J**) and Phospho-tau (**K**) induced by 10 µM Aβ_1–42_ alone. All results, which are presented as means  ±  SEM, were obtained from three to five separate experiments. **p  < 0.01, ***p  < 0.001

In keeping with earlier data [11, 42, 49, 58, 59], we showed that exposure of mouse cortical cultured neurons to oligomeric Aβ_1–42_ over 24 h can induce toxicity in a concentration-dependent manner, as evident from a reduction in MTT values (Fig. 10A). This was validated by a time-dependent increase in LDH levels in the conditioned media (Fig. 10B). Our DLS analysis revealed that unconjugated PLGA is quite stable in culture media over a 48 h period at 37 °C (Additional file 1: Fig. S3G–I). Subsequently, we showed that co-treatment of cultured neurons with 10 µM Aβ_1–42_ and 25 µM PLGA for 24 h was able to significantly protect neurons against toxicity (Fig. 10C, D). Additionally, we revealed that neurons treated with 10 µM Aβ_1–42_ + 25 µM PLGA incubated for 24 h at 37 °C prior to neuronal exposure can markedly increase neuronal viability when compared to treatment with 10 µM Aβ_1–42_ alone (Fig. 10E, F). In parallel, we noted that Aβ fibers disassembled following 72 h treatment with PLGA at 37 °C could significantly increase viability of cultured neurons compared to unaltered Aβ fibers (Fig. 10G–H). The protective effects of PLGA against Aβ-induced toxicity are accompanied by an attenuation of GSK-3β, ERK1/2 activation and decreased levels of phosphorylated tau at specific sites (Fig. 10I–K). These results, taken together, indicate that suppression of spontaneous Aβ aggregation or disassembly of aggregated Aβ fibers by PLGA can protect neurons against Aβ_1–42_-induced toxicity.

### PLGA-mediated clearance of labelled Aβ in adult mouse brain

**Fig. 11:**
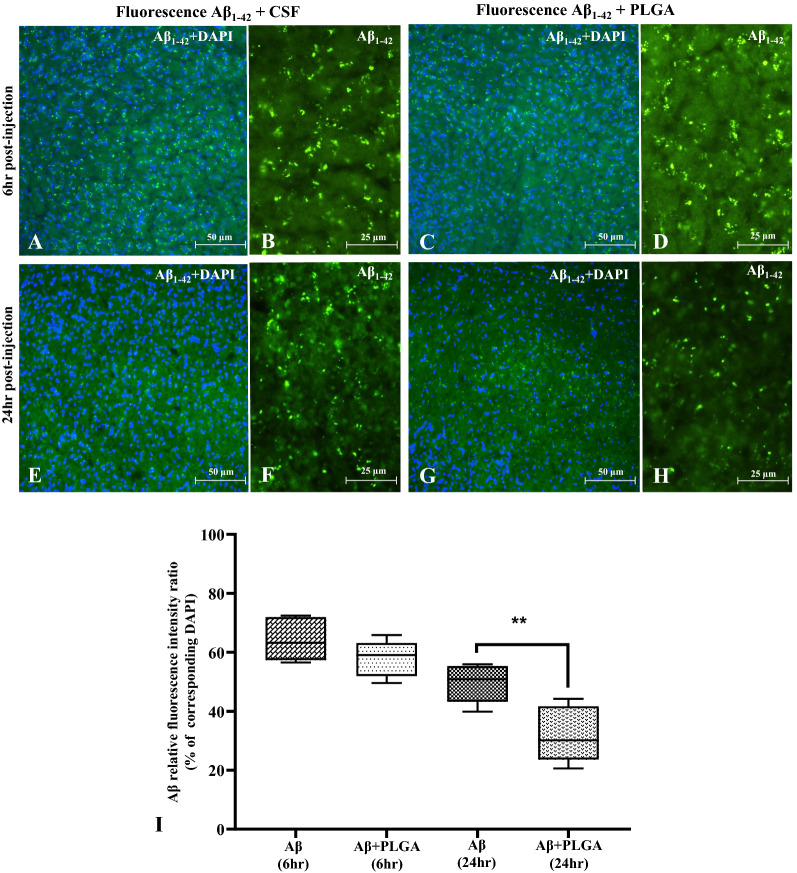
PLGA facilitates breakdown/clearance of injected Aβ in mouse brain. Photomicrographs depicting injected fluorescein labelled (green) Aβ_1–42_ peptide with (**A**, **C**, **E**, **G**) or without (**B**, **D**, **F**, **H**) DAPI at 6 h (**A**–**D**) and 24 h (**E**–**H**) in the cortex of CSF- (**A**, **B**, **E**, **F**) and PLGA (**C**, **D**, **G**, **H**)-injected mice. Note the decreased level of fluorescein labelled Aβ_1–42_ peptide in PLGA-treated brain slices at 24 h (**F**, **H**), but not at 6 h (**B**, **D**), compared to CSF-treated brain slices. **I** Histograms showing the quantification of Aβ relative fluorescence intensity ratio as percentage corresponding to their DAPI intensities at 6 h and 24 h in the cortex of CSF- and PLGA-injected mice brain tissue slices. Note the significant decrease of Aβ fluorescence intensity at 24 h, but not at 6 h, in mice injected with PLGA compared to CSF-injected mice. All data expressed as mean  ±  SEM were obtained from the quantifications of three to five zones of tissue section from each group. CSF, artificial cerebrospinal fluid. **p  <  0.01

To establish if native PLGA can influence the breakdown/clearance of Aβ_1–42_ in the brain, we injected fluorescence labelled Aβ_1–42_ with or without PLGA into the cortical region of mouse brains (Additional file 1: Fig. S4C–E) and then measured intensity of Aβ fluorescence at 6 and 24 h post-injection period. Fluorescence labelled Aβ_1–42_ was apparent both at 6 and 24 h after injection in the cortical regions away from the site of injection. Quantification of Aβ fluorescence intensity relative to DAPI showed a significant decline at 24 h, but not at 6 h, in the cortex of Aβ  +  PLGA treated mouse brains compared to mice treated with Aβ alone (Fig. 11A–I) suggesting a potential role for PLGA in the breakdown followed by clearance of Aβ peptides. However, these data need to be validated through further study.

## Discussion

The present study using a variety of experimental approaches revealed that FDA-approved biodegradable PLGA nanoparticles can attenuate temperature-dependent Aβ_1–42_ aggregation and protect neurons against Aβ-induced toxicity. This is supported by data which show that: (i) the rate of spontaneous Aβ_1–42_ aggregation increases with a rise in temperature from 27 to 40 °C, (ii) native PLGA of 50:50 resomer compared to other polymorphic nanoparticles such as PEG-PLGA and PCL potently inhibits Aβ aggregation at all studied temperatures partly by influencing a conformational shift from α-helix to β-sheet structure, (iii) PLGA triggers disassembly of matured Aβ_1–42_ fibers at a somewhat faster rate at 40 °C than 27 °C, (iv) PLGA-treated Aβ samples increase neuronal viability against Aβ toxicity by regulating tau phosphorylation and (v) native PLGA influences the breakdown/clearance of Aβ peptide in the brain. These results, taken together, suggest that native PLGA can inhibit Aβ aggregation and trigger disassembly of aggregated Aβ at temperatures outside the physiological range and can protect neurons against Aβ toxicity thus suggesting its unique therapeutic potential in the treatment of AD pathology.

It is accepted that monomeric Aβ exists as an unstructured random-coil, whereas fibrillar forms displays a characteristic cross-β structure with stacking of β strands perpendicular to the long fiber axis [60–62]. The intramolecular hydrophobic interaction involving amino acid sequence 17–24 (i.e., KLVFFAED) plays a critical role in the formation/stabilization of cross β-sheet structure. Conversion of monomeric random coil structured Aβ to β-sheet rich fibers occurs in three distinct phases: the lag phase for nucleation, the growth/linear phase for elongation and the plateau phase associated with aggregation/precipitation of fibrils. The formation of an ordered nucleus is the rate-limiting step in the fibrillization process during which Aβ aggregates are usually absent. Our results showed that the rate of Aβ_1–42_ fibril formation increased with peptide concentrations and rise in temperature from 27 to 40 °C as monitored by ThT kinetic assay and fluorescence imaging. The duration of the lag phase is reduced as a function of peptide concentration and temperature, whereas the fibril elongation phase is decreased with the rise of temperature at a given peptide concentration, possibly due to enhanced molecular collision facilitating hydrophobic interactions involved in the formation of Aβ fibrils. The subsequent plateau phase, on the other hand, escalated with peptide concentration but not much with the rise in temperature—suggesting that earlier phases of kinetic reactions are more thermodynamically dependent than the plateau phase of Aβ fibrillogenesis. It is likely that temperature facilitates fibril formation but not the fibrillar content as observed by the ThT binding assays. This is consistent with the reported effects of temperature on Aβ aggregation and fibril growth demonstrating the importance of hydrophobic interactions underlying the β-sheet formation and aggregation of Aβ peptide [13, 16].

Although temperature has been shown to influence the kinetics of amyloid fibril formation [15, 16, 18], very little is known how the presence of a nanoparticle or β-sheet blocker can impact temperature-dependent amyloid fibrillation. Certain gold nanoparticles have shown to increase Aβ aggregation at high temperature [63], whereas silica (hydrophilic) and polystyrene (hydrophobic) nanoparticles are found to differentially influence peptide aggregation depending on the temperature [18]. This is important for in vivo application of nanoparticles to treat AD-related amyloid pathology as the local temperatures in the brain can vary noticeably [18, 27, 64]. PLGA encapsulated drugs/agents have previously shown to inhibit Aβ aggregation and toxicity, but the effects have been attributed mostly to the interaction of the drugs with Aβ peptide rather than to PLGA [33, 40, 65–70]. Recently, we reported that PLGA without conjugation with any drug/agent can suppress Aβ fibrillization at normal physiological temperature (i.e., 37 °C), leading to the generation of shorter/fragmented fibrils [43]. The present result using ThT kinetic assays reveals that unconjugated PLGA of 50:50 resomer, but not 75:25 resomer, can supress temperature dependent spontaneous Aβ fibrilization outside of the physiological range. This is confirmed by fluorescence imaging, STEM and AFM showing the generation of shorter and fragmented fibrils/spherical globular aggregates as well as DLS analyses in which large fibrils that dominate the light scattering are reduced to smaller species. Furthermore, the suppression of Aβ_1–42_ aggregation by native PLGA was found to be strikingly more potent than some other polymorphic nanoparticles such as PEG-PLGA and PCL. The inhibition, which is evident as a function of PLGA concentration, is mediated not only by delaying the lag phase but also reducing the growth and saturation of Aβ fibrils during the respective linear and equilibrium phases—indicating that all three stages of Aβ fibrillogenesis are affected by native PLGA. This could be due to the interaction of PLGA with Aβ monomers precluding monomer–monomer hydrogen bonding and hydrophobic interactions preventing the development of critical nuclei and the elongation of fibrils. This is supported by liquid phase binding interaction as measured by ITC which showed an enthalpy driven (Δ*H*  < 0) binding between native PLGA and monomeric Aβ_1–42_. It is also possible that PLGA could destabilize the initial assembly to form a β-sheet rich scaffold during nucleation leading to an equilibrium shift towards the monomeric phase, which may render the elongation process energetically unfavorable. The interaction of PLGA with monomers and/or the scaffold may prolong the lag phase kinetics. Additionally, PLGA can interact with on-pathway species in the elongation phase and slow down the kinetics as evident in our results. This could be due to chirality of PLGA, which triggers binding with Aβ oligomers in the elongation phase through π–π and hydrophobic interactions resulting in destabilization of on-pathway and formation of off-pathway intermediates containing a mixture of α-helical and β-sheet conformations. This is supported partly by our filter-trap analysis showing an attenuation of Aβ_1–42_ oligomer content following exposure to unconjugated PLGA as well as our CD data which showed that PLGA nanoparticles enable Aβ peptides to remain more in a non-fibrillar state as apparent by reduced β-sheet structures.

It is of interest to note that Aβ fibril formation though increased with temperature as reported in earlier studies [12, 13, 16], suppression of spontaneous Aβ aggregation by PLGA is found to be inversely proportional to the temperature, specifically at higher concentrations of the nanoparticles. This is apparent not only in the ThT kinetic assays but also in fluorescence imaging, STEM, AFM and DLS analyses. Considering an increase in β-sheet structure in CD spectroscopy with rise in temperature, it appears that native PLGA is more effective at 27 °C than 40 °C in preventing the conversion of monomeric Aβ to fibrillar aggregates. This could be either due to; (i) slower Aβ aggregation kinetics, (ii) increased binding with Aβ monomer and/or nuclei or (iii) attenuating formation of off-pathway oligomeric intermediates. If any of these phenomena is involved in the detection of higher populations of small globular aggregates observed at 27 vs 40 °C remains to be determined from future study.

Accompanying attenuation of spontaneous Aβ aggregation, we observed that unconjugated PLGA can trigger disassembly of pre-aggregated Aβ fibers at all temperatures over an 72 h incubation period. This is evident by our ThT kinetic assays, fluorescence imaging, STEM, DLS and filter-trap analyses. Evidence suggests that the hydrophobicity of the Aβ peptide increases once it undergoes aggregation forming oligomers and fibrils [11, 71]. Apart from the hydrophobic Lys_16_ to Ala_21_ domain certain other residues such as His_14_, Gln_15_, Ala_30_, Ile_31_, Met_35_ and Val_36_ are known to play a role in the Aβ oligomerization and fibril formation. The C terminal domain is also involved in the formation of the protofilament structures due to generation of a hydrophobic core between the residues Ile_41_ and Val_39_ with the adjacent fibril. The N terminal domain of a fibril close to the C terminal domain of another fibril may also induce the formation of a salt bridge through intermolecular interactions between Asp_1_ and Lys_28_—an interaction that stabilizes the interface between the two protofilaments [72, 73]. Our previous molecular docking analysis revealed that PLGA can interfere with the salt bridges and with the residues present in the steric zipper domain—an interaction that can trigger disassembly of the preformed fibrillar structure [43]. Similar phenomena have been reported using a variety of phytochemicals, peptides and polymers including curcumin, clioquinol, epigallocatechin gallate, polyproline and myricetin [74–78]. Our results also revealed that intracerebral injection of unconjugated PLGA can attenuate the levels of administered fluorescently labelled Aβ peptide suggesting PLGA, as observed under in vitro condition, may influence breakdown followed by clearance of Aβ peptide under in vivo condition, though this needs to be validated from further experiments.

Apart from suppressing Aβ aggregation, we showed spontaneous inhibition of Aβ aggregation by PLGA can markedly increase viability of cultured cortical neurons. Disassembly of aggregated Aβ fibers by PLGA also found to attenuate cell toxicity compared to aggregated fibers. The protective effect is accompanied by reducing phosphorylation of tau protein and its associated signaling pathway. This is likely due to the retention of Aβ monomers or sequestration of Aβ peptides into amorphous aggregates, which are less toxic than metastable Aβ oligomers/aggregates [58, 71, 79, 80]. Since temperature-dependent changes are reversible [81] we evaluated the protective effect of PLGA treated Aβ samples incubated for 24 h at 37 °C—the temperature used in culturing/maintaining primary neurons. However, it would be of interest to determine if maintaining and treating neurons at different temperatures (i.e., 27, 37 and 40 °C) with PLGA treated Aβ samples incubated 24 h with the corresponding temperature can differentially affect neuronal viability. This will highlight the potential of PLGA to protect neurons against Aβ toxicity by inhibiting the formation or triggering the disassembly of higher-order aggregates.

At present, there is no effective treatment to prevent/arrest the progression of AD. The cholinesterase inhibitors and the glutamate NMDA receptor antagonist memantine that have been approved for the treatment provide symptomatic relief for only a fraction of AD patients [9, 82, 83]. The beneficial effects of recently approved disease modifying Aβ monoclonal antibody Aducanumab on AD patients remain to be determined [84, 85]. Some earlier studies have shown that PLGA nanoparticles functionalized with various drugs/agents such as donepezil, galantamine, quercetin, memantine and curcumin can exhibit beneficial effects on cellular and/or animal models of AD compared to cells/mice treated with drugs alone or vehicles used for dissolving drugs/PLGA nanoparticles [37, 39, 40, 67, 69, 70, 86, 87]. Interestingly, our recent study reveals that unconjugated PLGA nanoparticles by inhibiting Aβ aggregation/toxicity can also attenuate AD-related pathology in cellular and animal models of AD [43]. However, no study has evaluated if PLGA can influence Aβ aggregation kinetics at different temperatures which affect multiple facets of Aβ pathology [12, 16, 24, 62, 88, 89] and fluctuate from 33.4 to 42 °C in brain disease/pathological conditions [18, 27, 28]. Intriguingly, unconjugated PLGA, as apparent from this study, can inhibit spontaneous aggregation and trigger disassembly of aggregated Aβ at various temperatures (i.e., 27–40 °C). PLGA treated Aβ samples can also increase viability of cultured cortical neurons by regulating conformational state of Aβ peptide and its associated cellular signaling pathway involving site-specific phosphorylation of tau protein. Additionally, unconjugated PLGA was found to influence the breakdown/clearance of fluorescence labelled Aβ_1–42_ in the brain. Although these results need to be validated from further study, they highlight the significance of unconjugated PLGA in targeting Aβ peptide and its unique therapeutic potential in the treatment of AD.

## Supplementary Information

**Additional file 1: Figure S1.** ThT kinetic assays showing aggregation of 2.5–20 µM Aβ_1–42_ over a 24 h incubation at 27 °C (**A**), 37 °C (**B**) and 40 °C (**C**). **Figure S2. A**–**C** ThT assays showing aggregation kinetics (**A**, **D**, **G**) and respective fluorescence images of 10 µM Aβ_1–42_ (**B**, **E**, **H**) and 10 µM Aβ_42–1_ (**C**, **F**, **I**) over 24 h incubation at 27 °C (**A**–**C**), 37 °C (**D**–**F**) and 40 °C (**G**–**I**). Note the absence of Aβ_42–1_ aggregation at any temperature over 24 h incubation. **Figure S3.** DLS analysis depicting a peak of  ~ 100 nm diameter for unconjugated PLGA and its stability in phosphate buffer over 48 h at 27 °C (**A**, **B**), 37 °C (**C**, **D**) and 40 °C (**E**, **F**). DLS analysis depicting a peak of  ~ 100 nm diameter for PLGA in phosphate buffer (**G**) and its stability at 37 °C over 48 h in culture medium (**H**, **I**). Note that PLGA nanoparticles are quite stable both in the phosphohate buffer as well as in culture medium over 48 h period. **Figure S4.** Histograms showing protection of mouse cultured neurons following co-treatment of 10 µM Aβ_1–42_ with 5 µM PEG-PLGA (**A**) or 100 nM PCL (**B**) over 24 h compared to neurons treated with 10 µM Aβ_1–42_ as detected using MTT assay. **C**–**E** Mouse brain section showing the site of fluoresence Aβ_1-42_ injection (arrow) using Hamilton syringe under anesthesia. The brain section shows nuclear labelling with DAPI (**C**), presence of fluoresence Aβ_1–42_ (**D**) and the merged image (**E**).

## Data Availability

The data in this work are available in the manuscript or Additional file or available from the corresponding author upon reasonable request.

## Abbreviations

Aβ: Amyloid β
AD: Alzheimer’s disease
AFM: Atomic force microscopy
CD: Circular dichroism
DLS: Dynamic light scattering
DMEM: Dulbecco’s modified Eagle’s medium
DMSO: Dimethyl sulfoxide
FBS: Fetal bovine serum
HBSS: Hanks’ balanced salt solution
HFIP: Hexafluoro-2-propanol
ITC: Isothermal titration calorimetry
LDH: Lactate dehydrogenase
MTT: 3-(4,5-Dimethylthiazolyl)-2,5-diphenyltetrazolium bromide
PBS: Phosphate-buffered saline
PCL: Polycaprolactone
PLGA: Poly(d,l-lactide-co-glycolide)
PEG-PLGA: Polyethylene glycol-PLGA
STEM: Scanning transmission electron microscopy
ThT: Thioflavin-T
