# Supplementary figures and images for: Unconjugated PLGA nanoparticles attenuate temperature-dependent β-amyloid aggregation and protect neurons against toxicity: implications for Alzheimer’s disease pathology
Source: J Nanobiotechnology. 2022 Feb 4;20:67. doi: 10.1186/s12951-022-01269-0 (PMC8817552; doi:10.1186/s12951-022-01269-0)

# Supplementary Figure 1

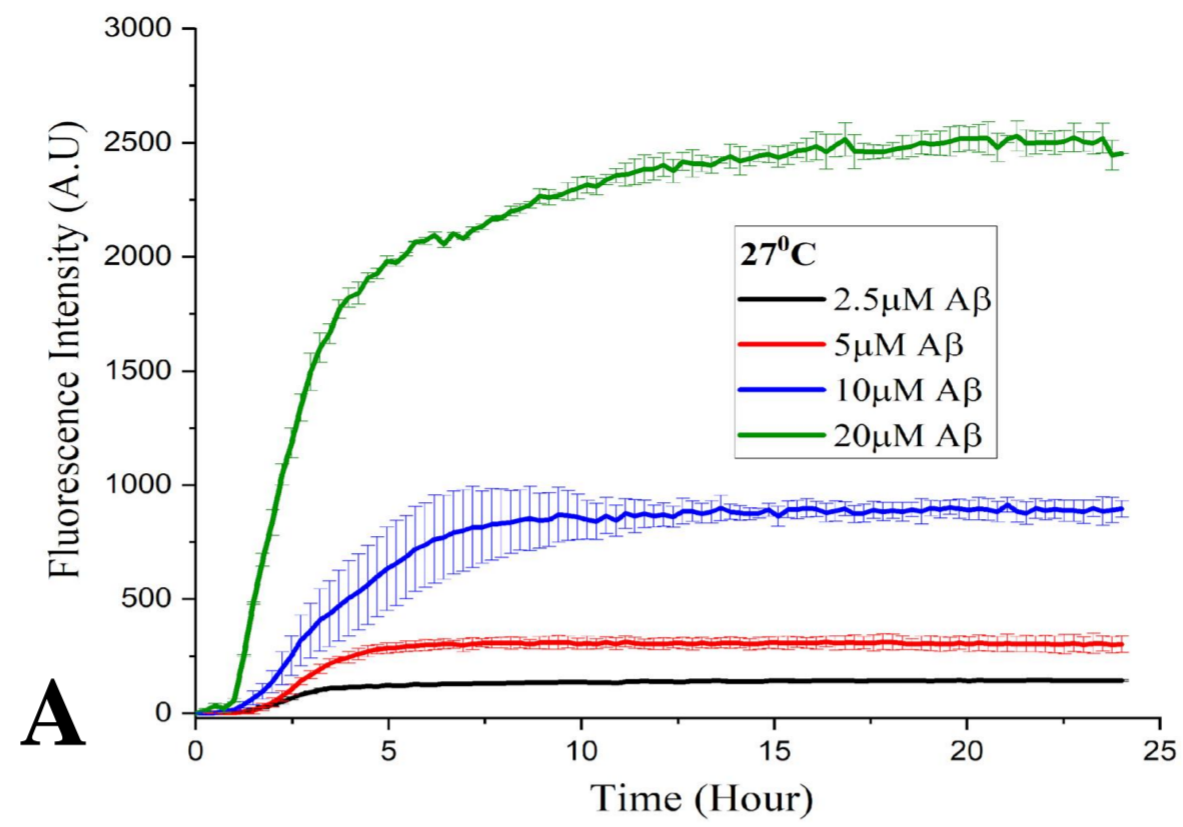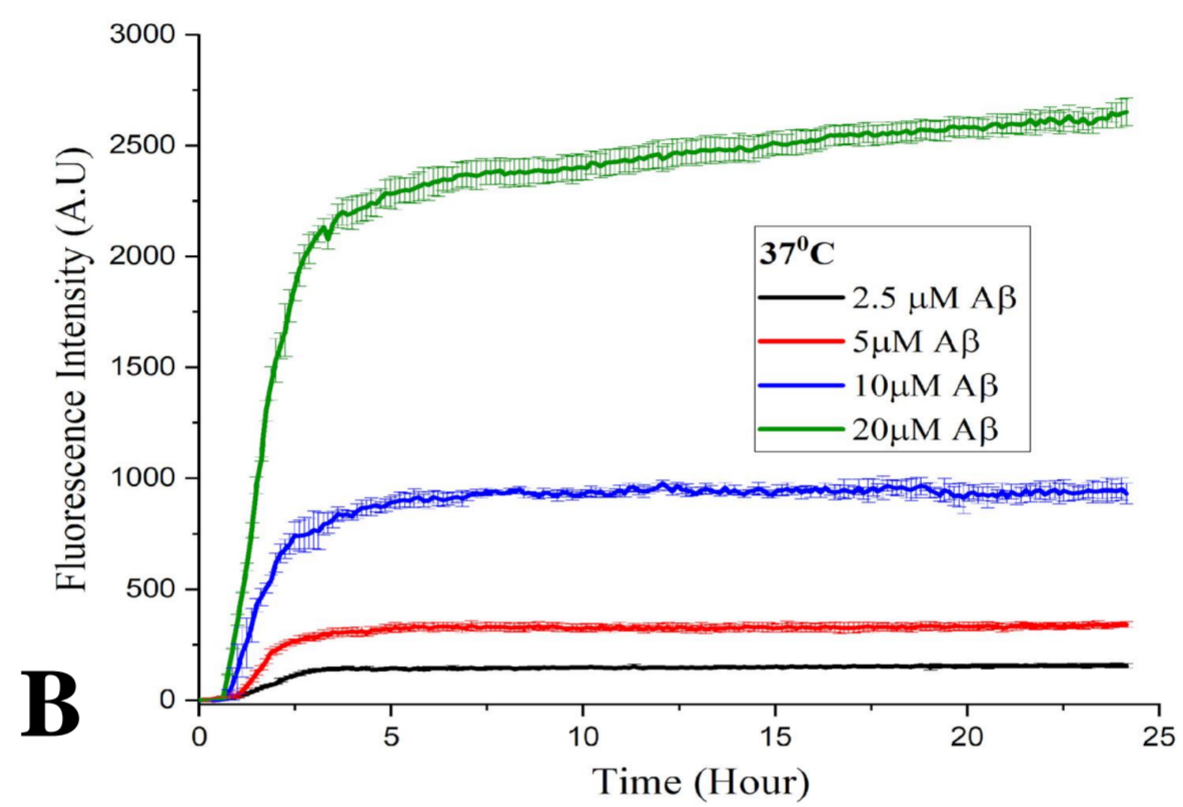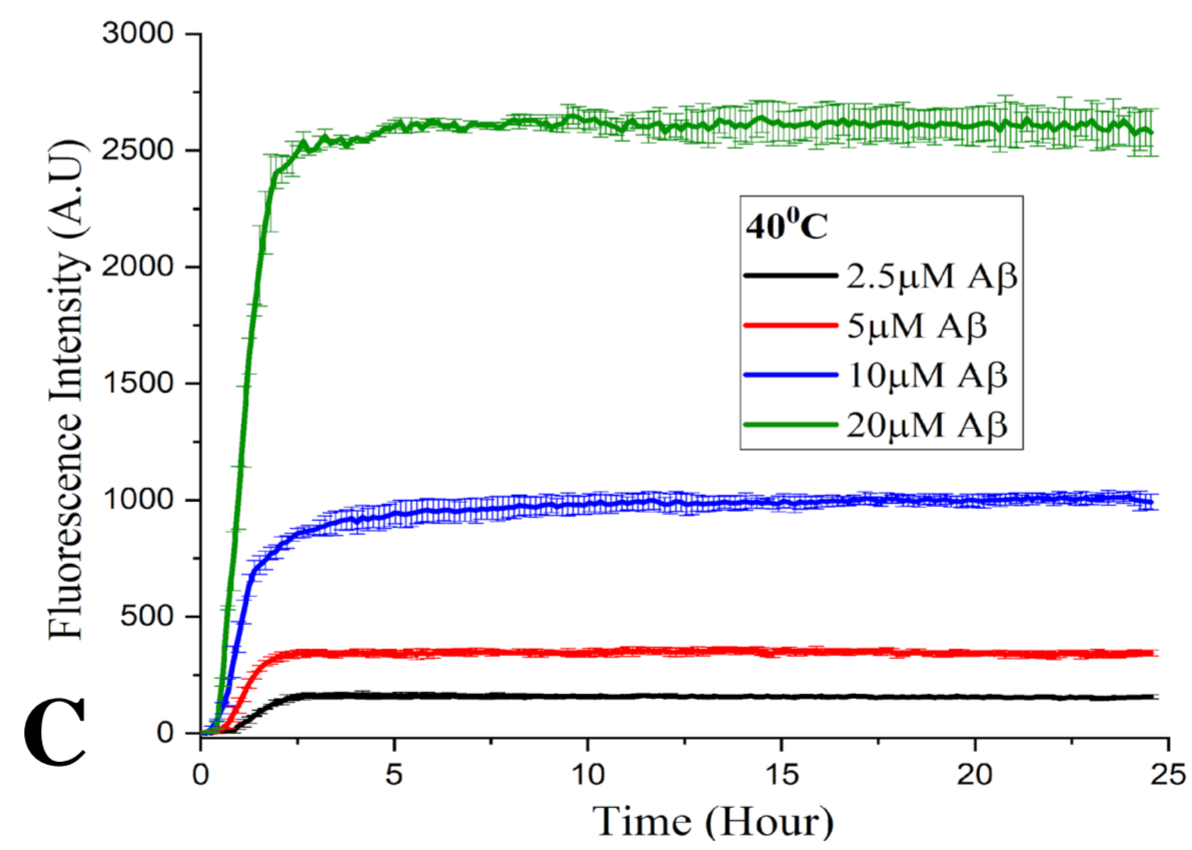

Supplementary Figure 2

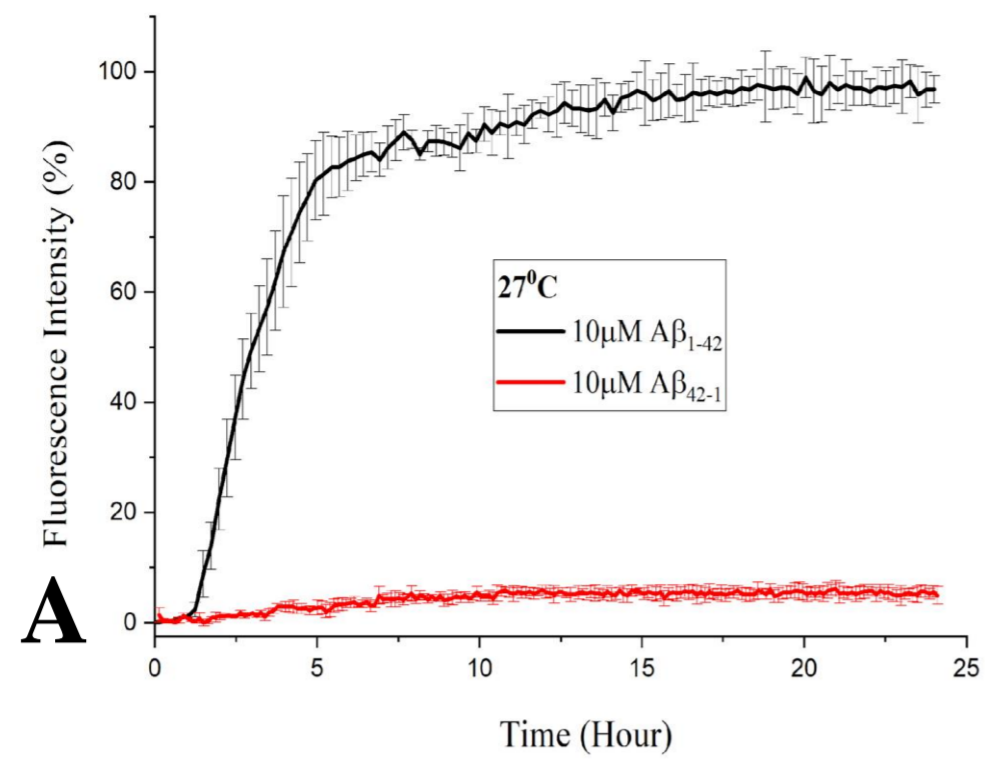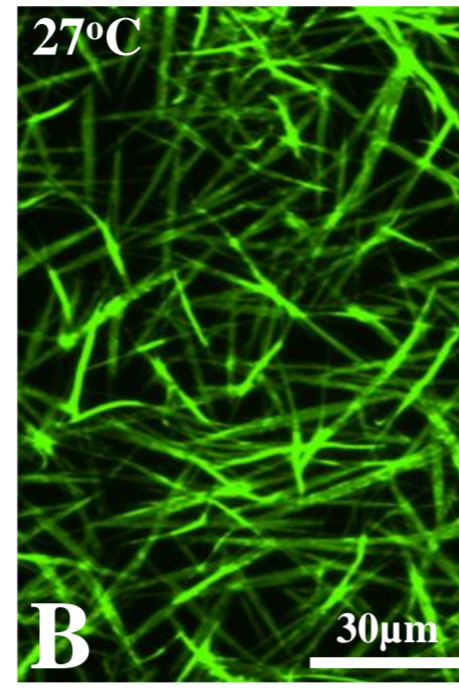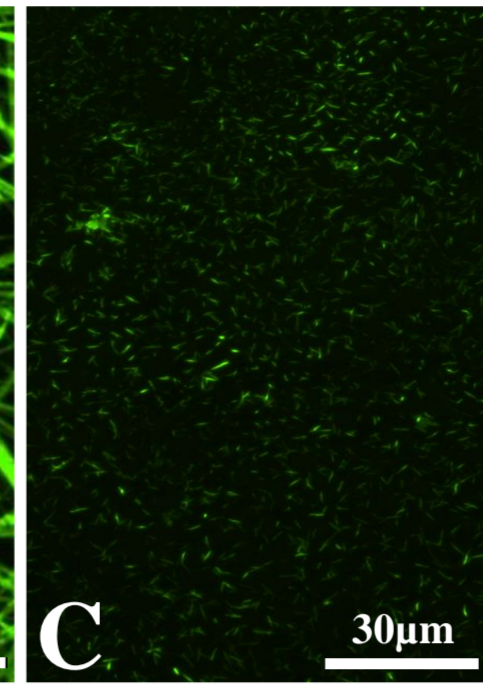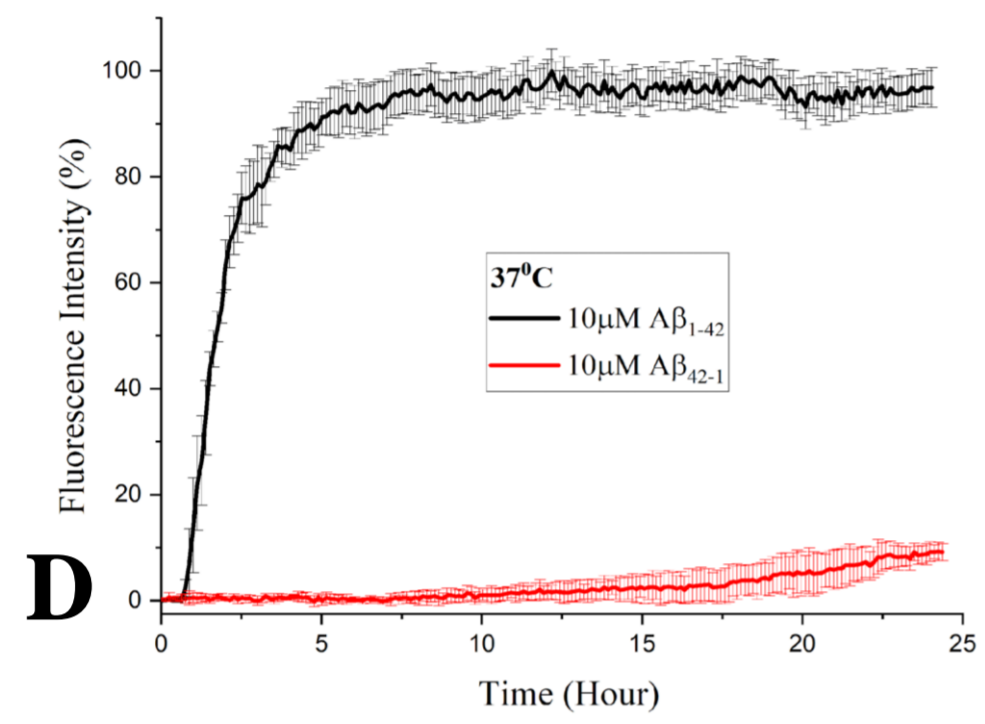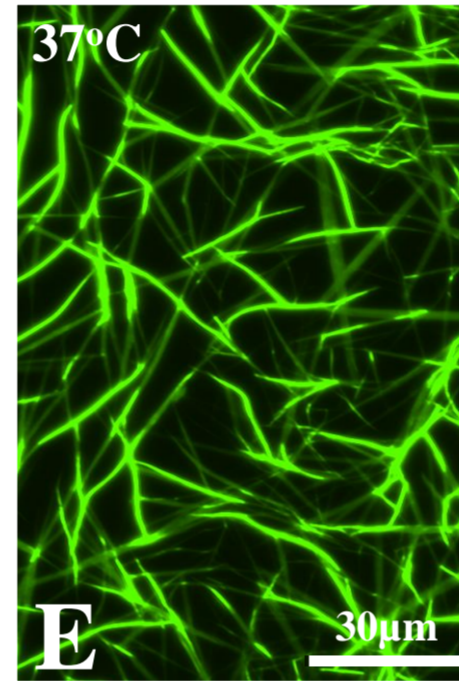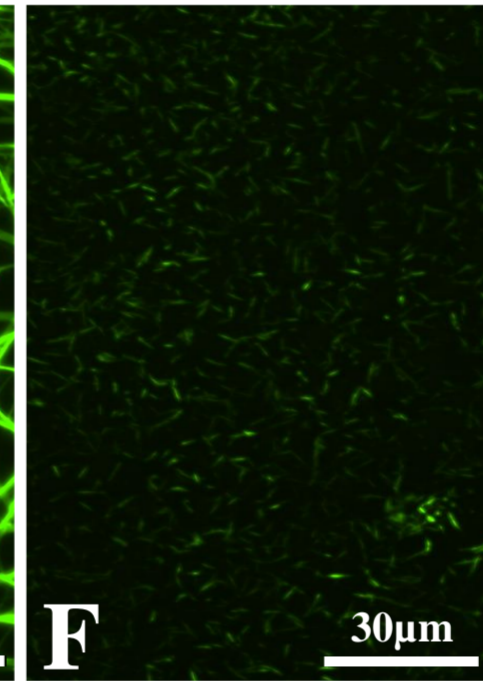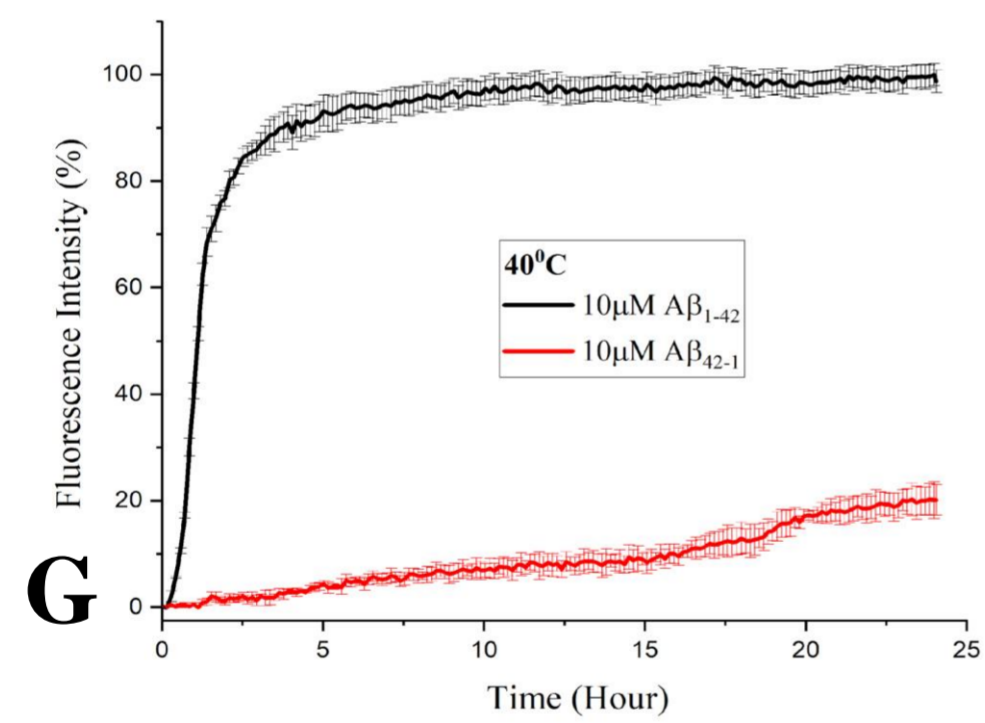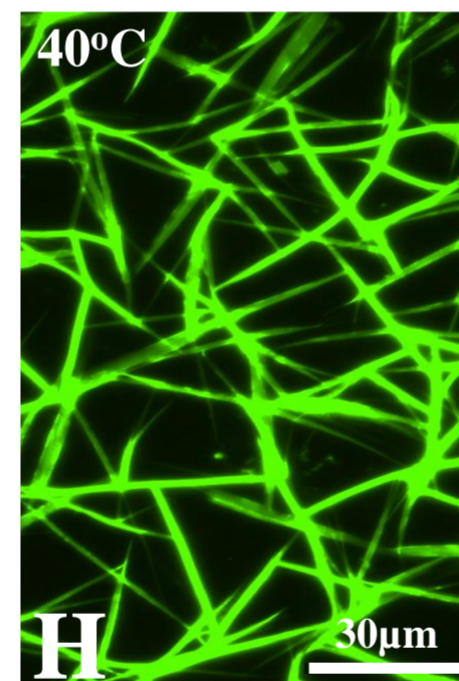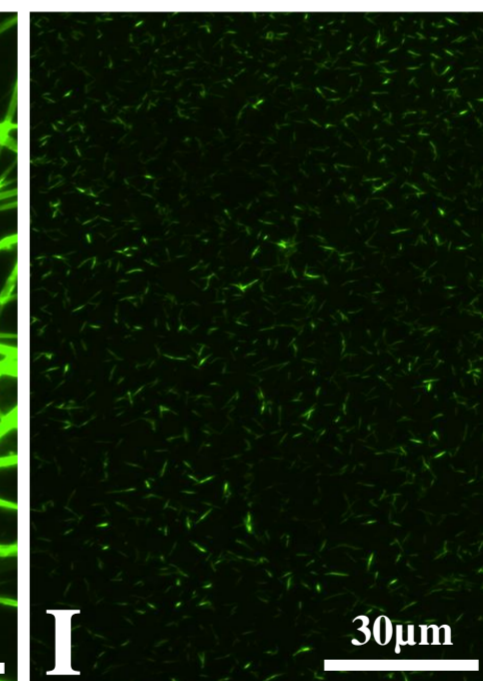

# Supplementary Figure 3

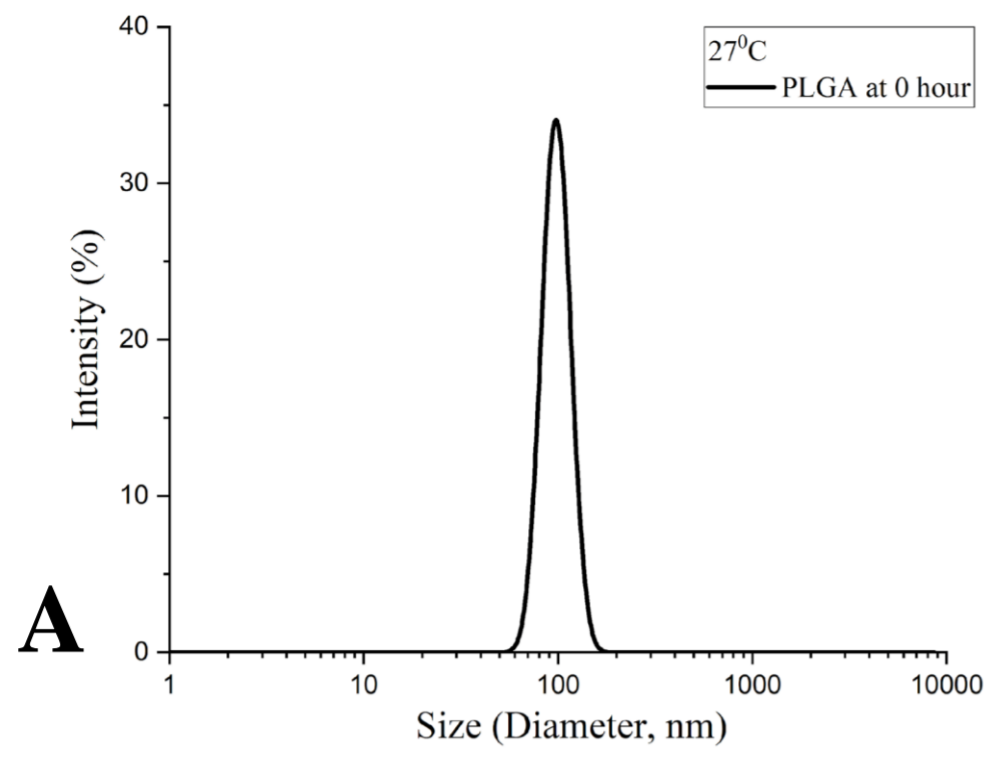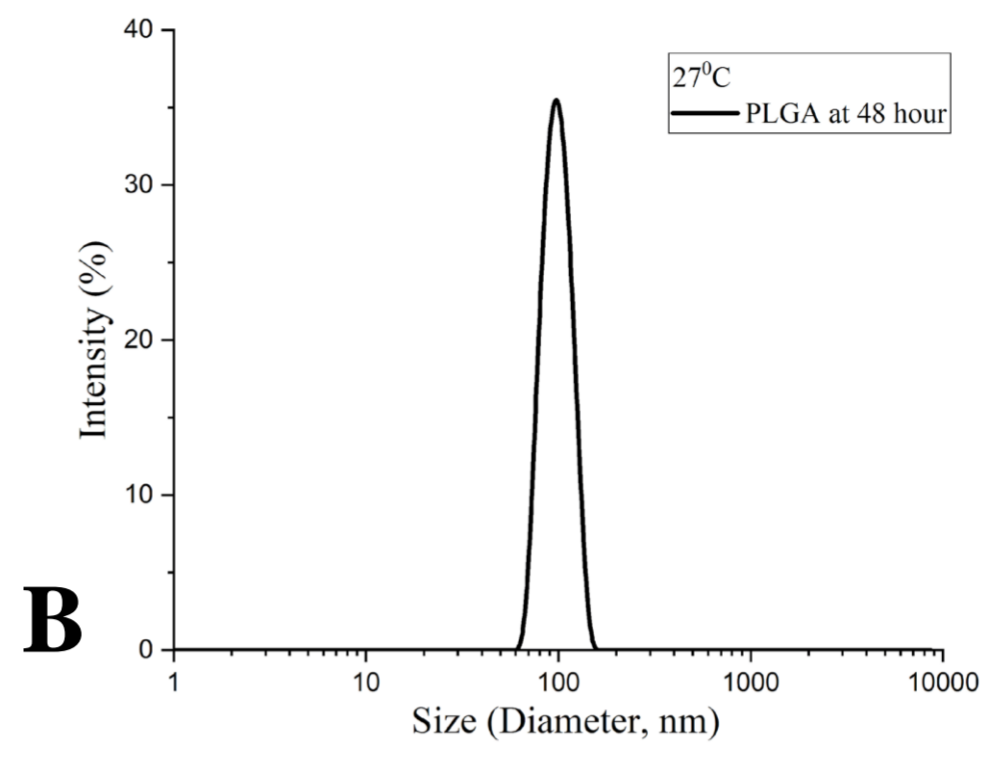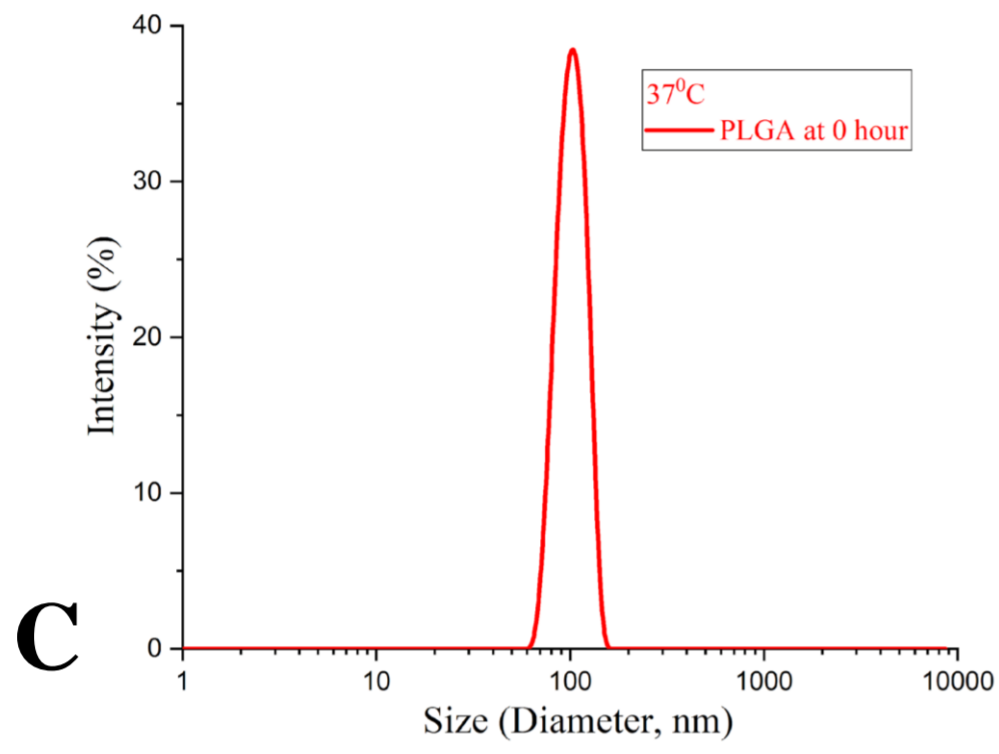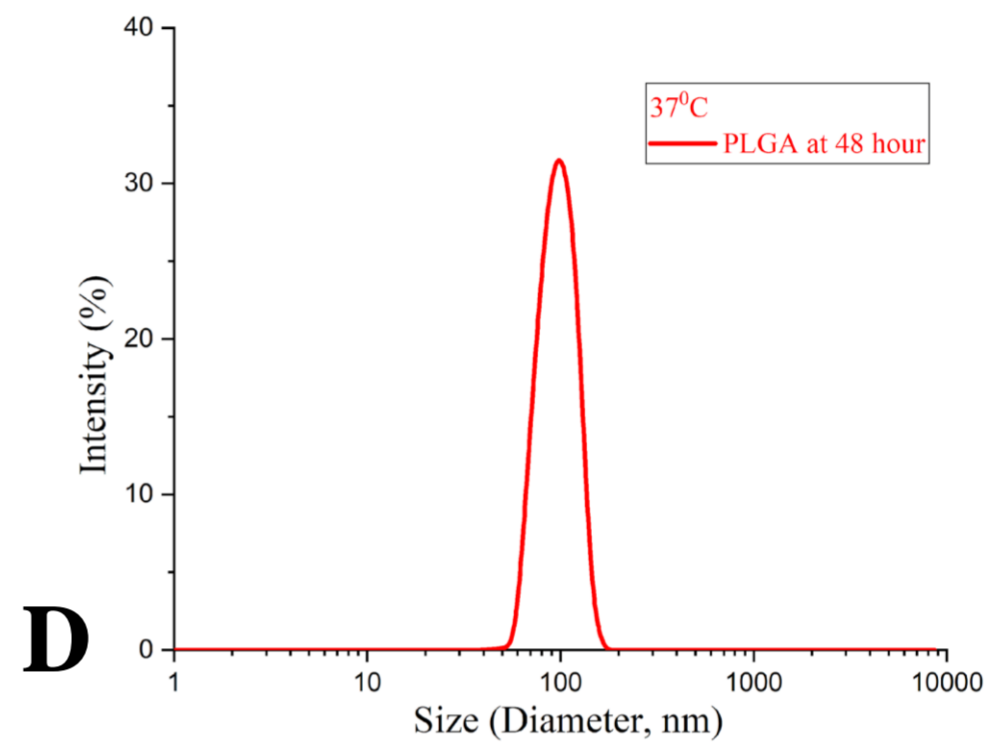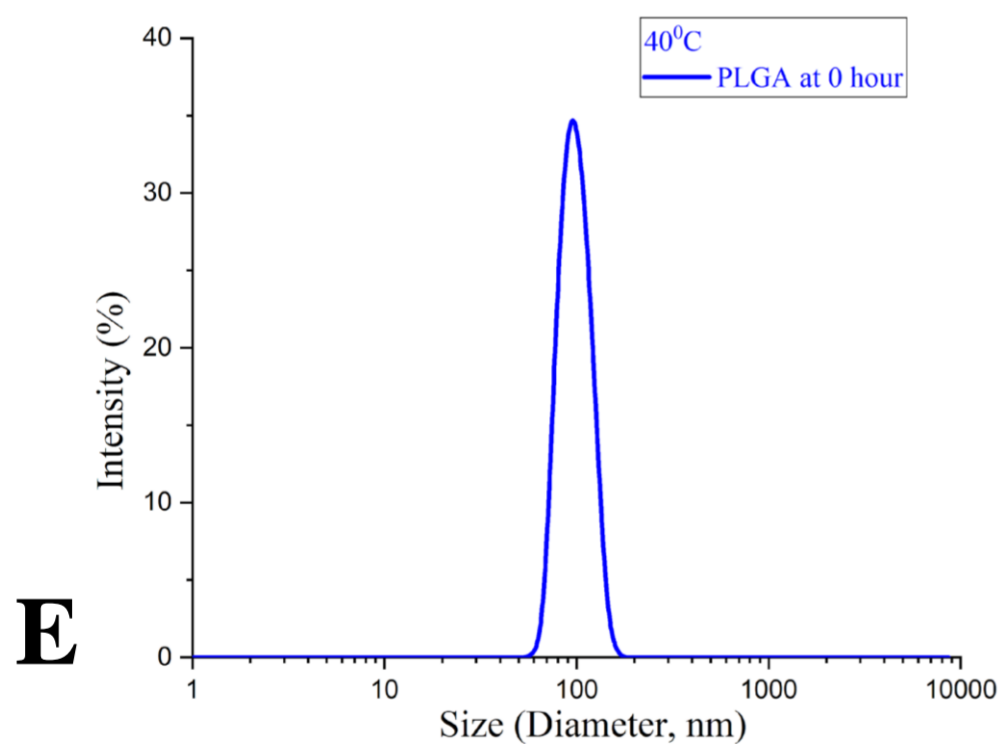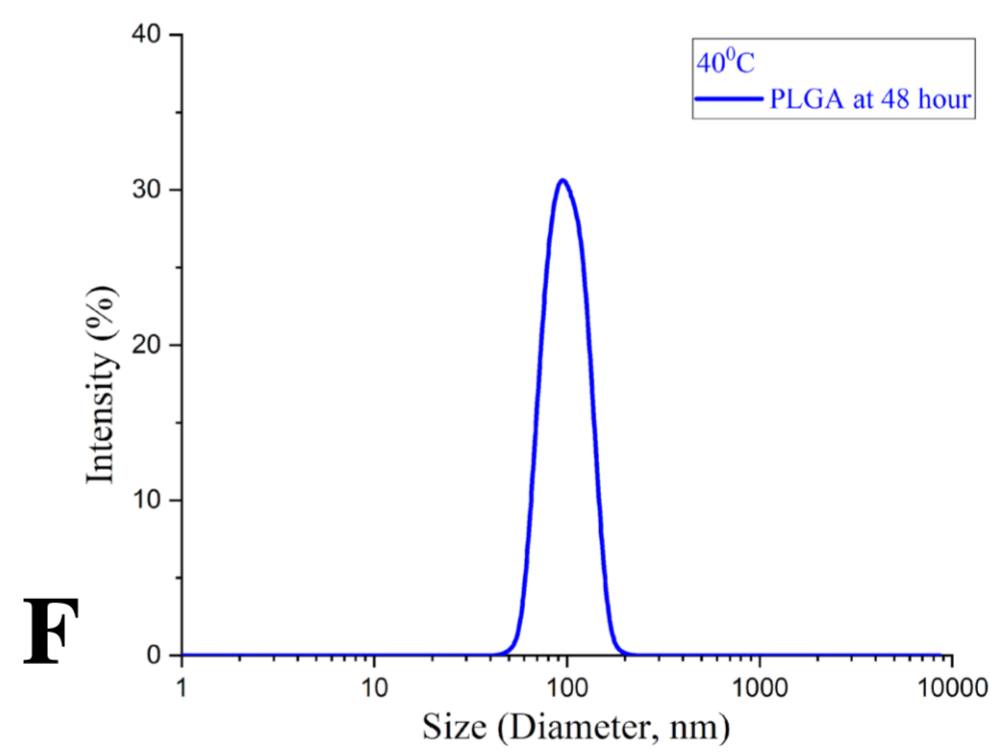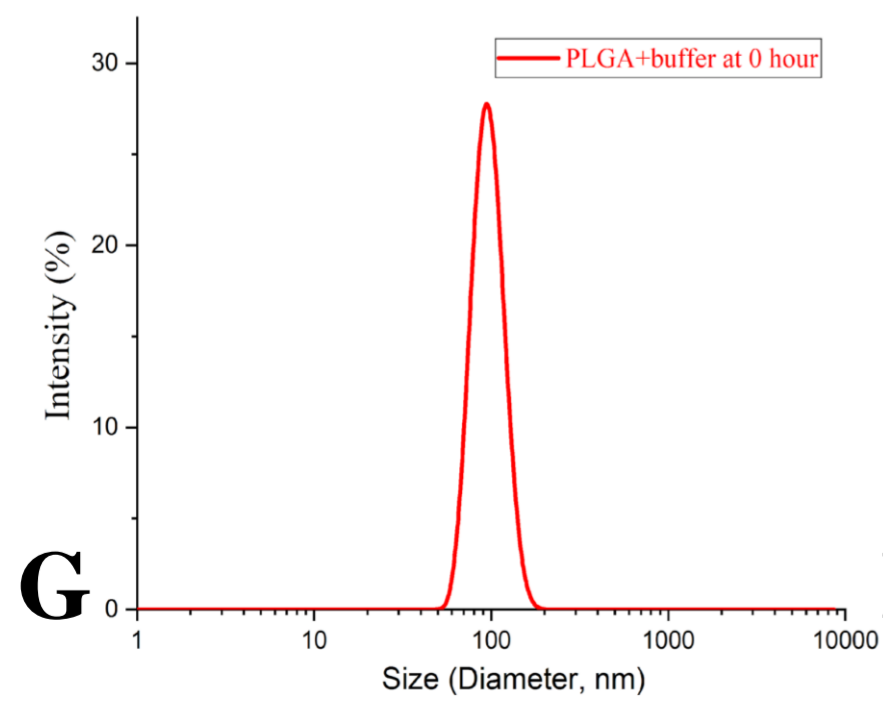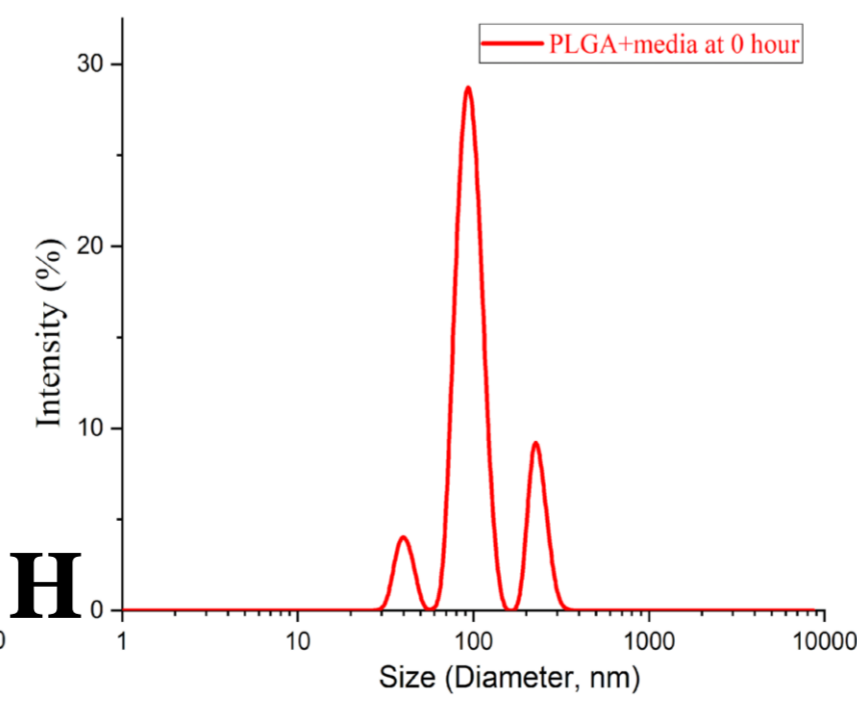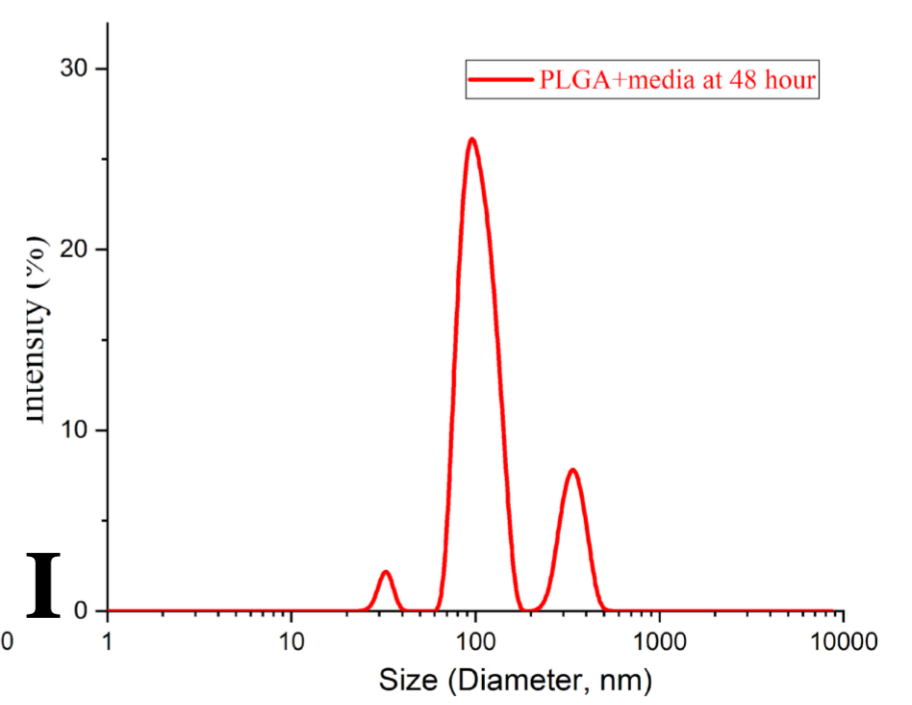

Supplementary Figure 4

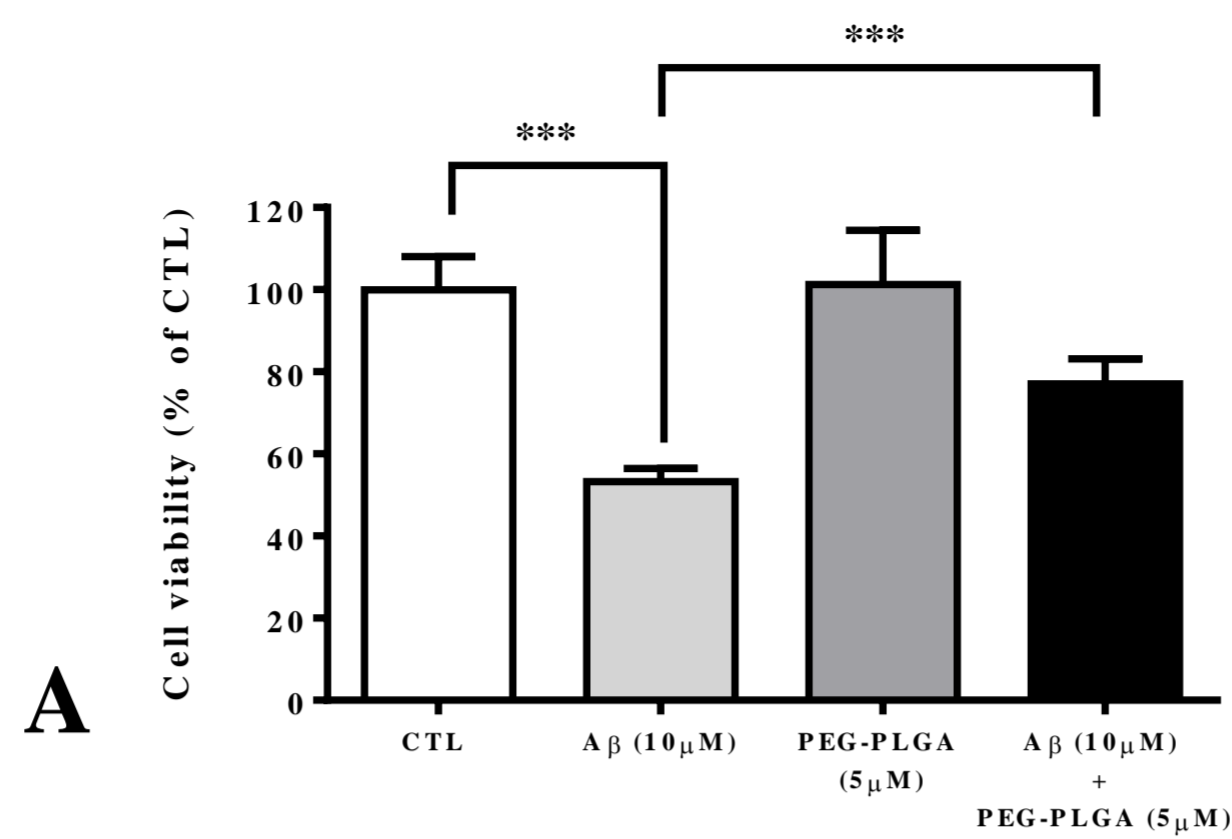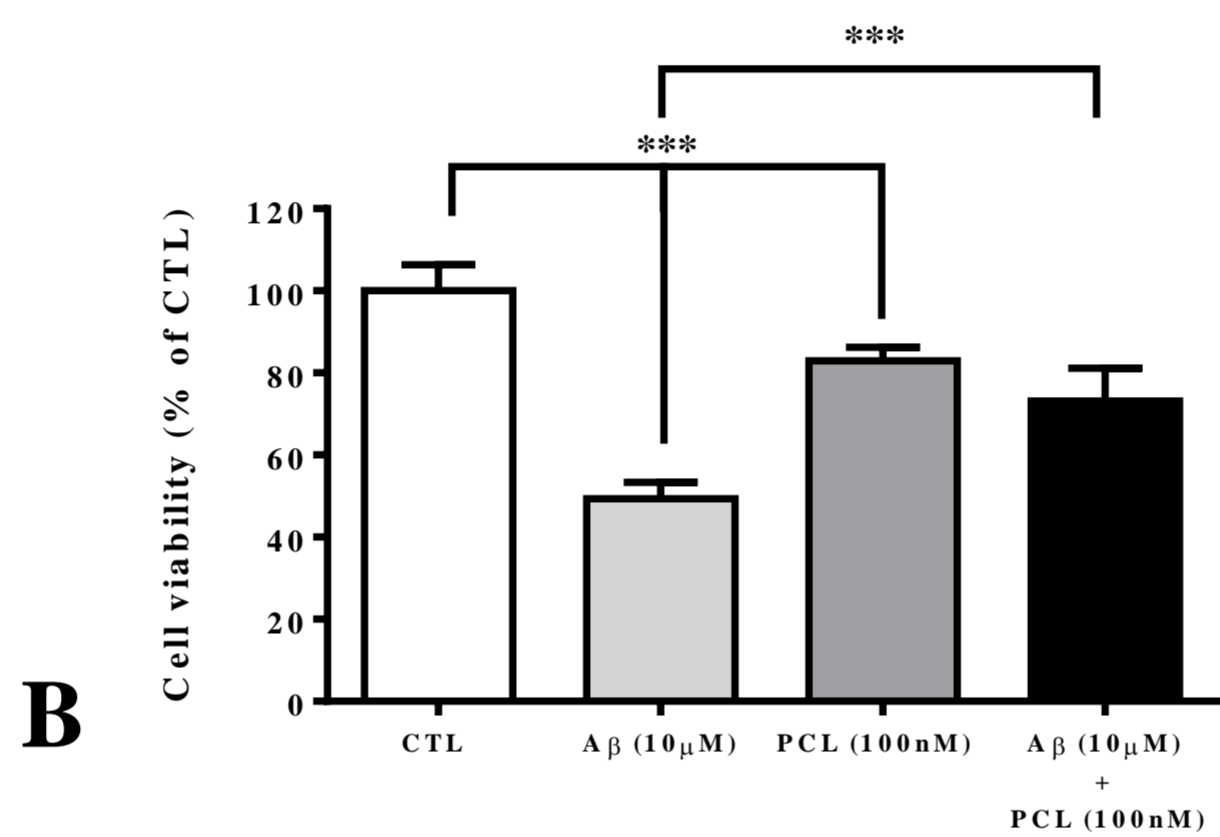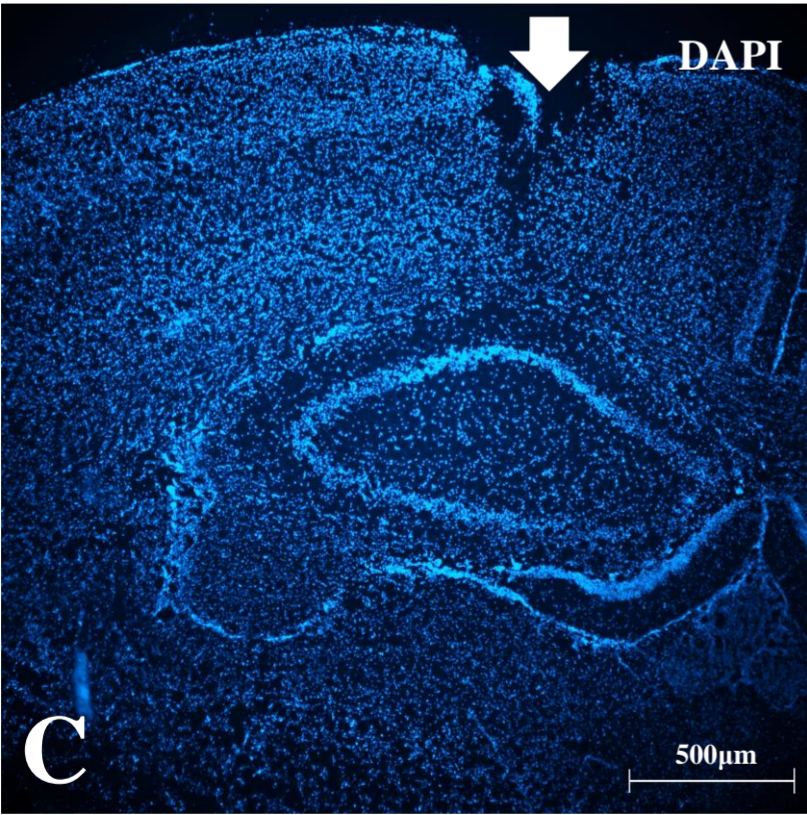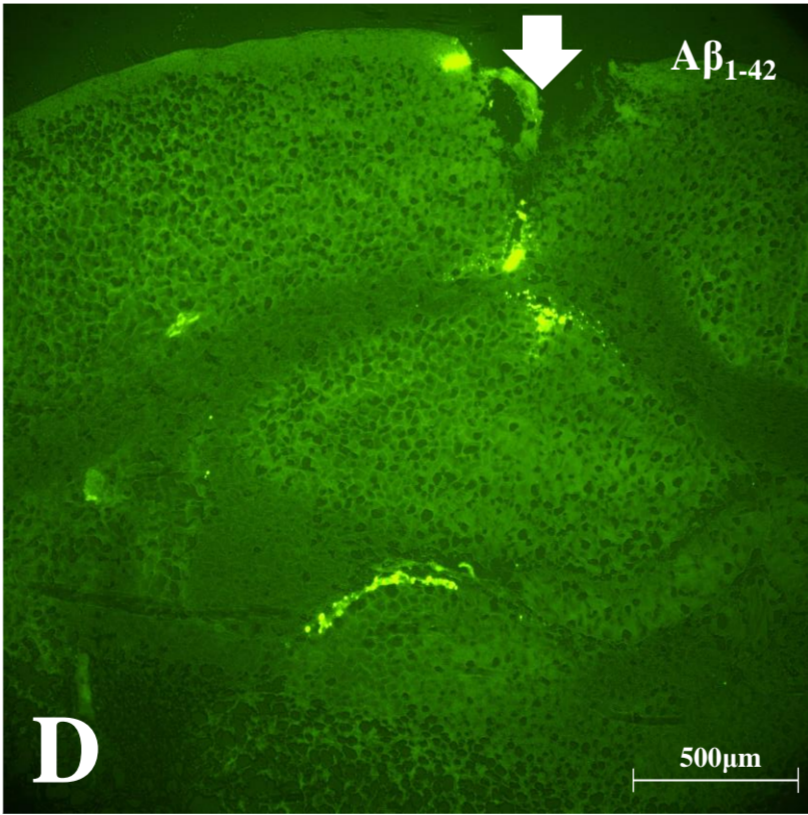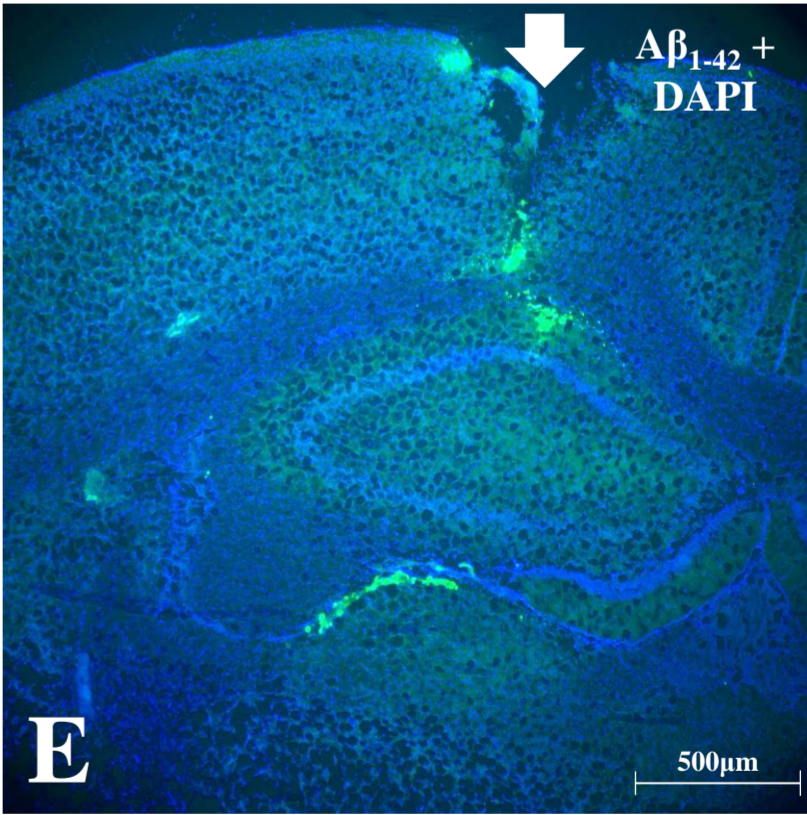

Supplement: Supplementary file 1 — Additional file 1: Figure S1. ThT kinetic assays showing aggregation of 2.5–20 µM Aβ1–42 over a 24 h incubation at 27 °C (A), 37 °C (B) and 40 °C (C). Figure S2. A–C ThT assays showing aggregation kinetics (A, D, G) and respective fluorescence images of 10 µM Aβ1–42 (B, E, H) and 10 µM Aβ42–1 (C, F, I) over 24 h incubation at 27 °C (A–C), 37 °C (D–F) and 40 °C (G–I). Note the absence of Aβ42–1 aggregation at any temperature over 24 h incubation. Figure S3. DLS analysis depicting a peak of ~ 100 nm diameter for unconjugated PLGA and its stability in phosphate buffer over 48 h at 27 °C (A, B), 37 °C (C, D) and 40 °C (E, F). DLS analysis depicting a peak of ~ 100 nm diameter for PLGA in phosphate buffer (G) and its stability at 37 °C over 48 h in culture medium (H, I). Note that PLGA nanoparticles are quite stable both in the phosphohate buffer as well as in culture medium over 48 h period. Figure S4. Histograms showing protection of mouse cultured neurons following co-treatment of 10 µM Aβ1–42 with 5 µM PEG-PLGA (A) or 100 nM PCL (B) over 24 h compared to neurons treated with 10 µM Aβ1–42 as detected using MTT assay. C–E Mouse brain section showing the site of fluoresence Aβ1-42 injection (arrow) using Hamilton syringe under anesthesia. The brain section shows nuclear labelling with DAPI (C), presence of fluoresence Aβ1–42 (D) and the merged image (E). [file 12951_2022_1269_MOESM1_ESM.pdf]
